# Crystal structures of three mercury(II) complexes [HgCl_2_ *L*] where *L* is a bidentate chiral imine ligand

**DOI:** 10.1107/S2056989015020368

**Published:** 2015-11-07

**Authors:** Guadalupe Hernández, Sylvain Bernès, Oscar Portillo, Alejandro Ruíz, Gloria E. Moreno, René Gutiérrez

**Affiliations:** a Laboratorio de Síntesis de Complejos, Facultad de Ciencias Químicas, Universidad Autónoma de Puebla, A.P. 1067, 72001 Puebla, Pue., Mexico; b Instituto de Física, Universidad Autónoma de Puebla, Av. San Claudio y 18 Sur, 72570 Puebla, Pue., Mexico; c Departamento de Microbiología, Facultad de Ciencias Químicas, Universidad Autónoma de Puebla, Puebla, Pue., Mexico

**Keywords:** crystal structure, mercury, Schiff base, disphenoidal geometry

## Abstract

The crystal structures of three complexes [HgCl_2_ *L*] were determined, namely, (*S*)-(+)-di­chlorido­[1-phenyl-*N*-(pyridin-2-yl­methyl­idene)ethyl­amine-κ^2^ *N*,*N*′]mercury(II), [HgCl_2_(C_14_H_14_N_2_)], (*S*)-(+)-di­chlorido­[1-(4-methyl­phen­yl)-*N*-(pyridin-2-yl­methyl­idene)ethyl­amine-κ^2^ *N*,*N*′]mercury(II), [HgCl_2_(C_15_H_16_N_2_)], and (1*S*,2*S*,3*S*,5*R*)-(+)-di­chlorido­[*N*-(pyridin-2-yl­methyl­idene)isopino­camph­eyl­amine-κ^2^ *N*,*N*′]mercury(II), [HgCl_2_(C_16_H_22_N_2_)]. The complexes consist of a bidentate chiral imine ligand coordinating to HgCl_2_ and crystallize with four independent mol­ecules in the first complex and two independent mol­ecules in the other two. The coordination geometry of mercury is tetra­hedral, with strong distortion towards a disphenoidal geometry, as a consequence of the imine bite angle being close to 70°. The Cl—Hg—Cl angles span a large range, 116.0 (2)–138.3 (3)°, which is related to the aggregation state in the crystals. For small Cl—Hg—Cl angles, complexes have a tendency to form dimers, *via* inter­molecular Hg⋯Cl contacts. These contacts become less significant in the third complex, which features the largest intra­molecular Cl—Hg—Cl angles.

## Abstract 2

Three complexes synthesized by coordination of chiral imines to HgCl_2_ have been characterized, in which the tetra­hedral Hg^II^ centre has a geometry strongly distorted towards the disphenoidal geometry.

## Chemical context   

The coordination geometry for Hg^II^ is very versatile, in particular because the available coordination numbers for this 5*d* ^10^ metal ion cover a large range, from 2 (*e.g*. Moreno-Alcántar *et al.*, 2013 ▸) to 10 (Williams *et al.*, 2009 ▸). In the case of tetra­coordinated Hg^II^ complexes, the possible geometry extends from square planar, similar to *d* ^8^ transition metals, to tetra­hedral, as for *d* ^7^ transition metals. Inter­mediate situations resulting from a distortion of the tetra­hedral geometry are, however, the most common. The disphenoidal arrangement, also known as a *seesaw geometry*, is frequently observed in mononuclear Hg^II^ complexes bearing non-sterically demanding ligands with significant σ-donating ability. This geometry, resulting from the formal distortion *T*_d_ → *C*_2v_, may be regarded as derived from a trigonal bipyramid, with an unoccupied site in the equatorial plane (*e.g*. Bell *et al.*, 1988 ▸; Wang *et al.*, 2005 ▸). Much less frequently observed is the symmetry distortion *T*_d_ → *C*_3v_, for which one axial site of the trigonal bipyramid is vacant (*e.g*. Adams *et al.*, 1970 ▸).

Within this class of complexes, the coordination of the HgCl_2_ mol­ecule to a Schiff base is of inter­est, especially if the donor atoms from the ligand form a bite angle on the metal. Since this angle is generally less than 90°, a substantial distortion of the *T*_d_ geometry is expected, which could modulate inter­molecular inter­actions in the crystal.

We gained experience in the synthesis of such ligands *via* sustainable processes, using solvent-free one-pot reactions between a chiral amine and an aldehyde, providing that at least one reactant is liquid at room temperature. Three Schiff bases in this series, synthesized from 2-pyridine­carboxaldehyde, have been coordinated to HgCl_2_, and we now report the crystal structures of the resulting complexes. The main purpose of the X-ray characterization is to assess the consequence of the N—Hg—N bite angle on the coordination geometry. Moreover, the synthetic chemistry of Hg^II^ compounds is still topical, mainly due to their potential applications as electroluminescent devices (Fan *et al.*, 2009 ▸), sensors (Zhou *et al.*, 2010 ▸), fluorescent lamps, batteries and preservatives in wood-pulp industry, *etc*. The inter­ference of this metal in biological systems, mainly by targeting and eventually inactivating thio-containing enzymes, also requires a better understanding of its coordinative properties (Shettihalli & Gummadi, 2013 ▸).
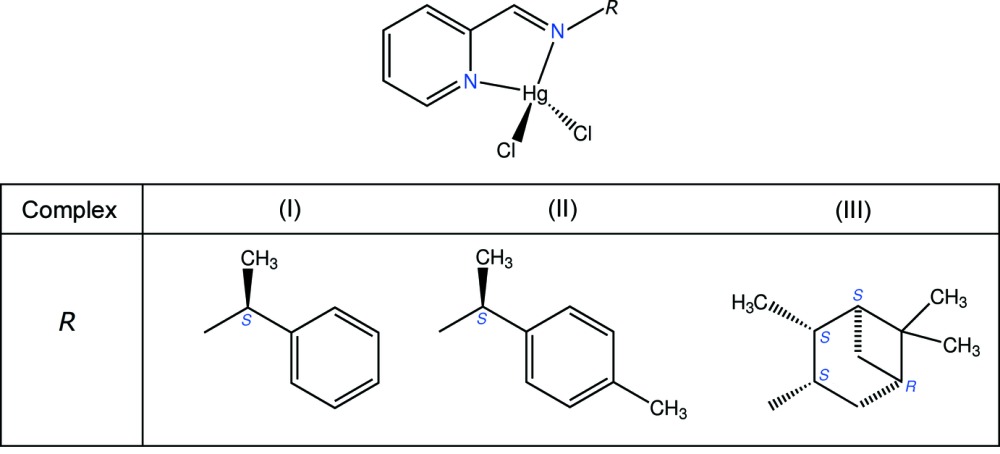


## Structural commentary   

**Figure 1:**
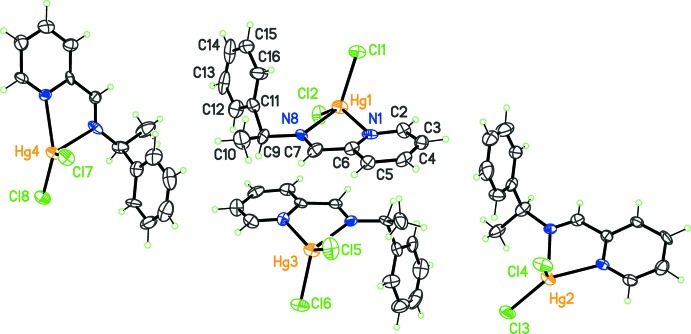
The asymmetric unit for complex (I), with displacement ellipsoids at the 30% probability level. The labels for C and N atoms in mol­ecules Hg2, Hg3 and Hg4 are as in mol­ecule Hg1, but increased by 20, 40 and 60, respectively.

The first imine, *L* ^1^, was obtained by condensation between 2-pyridine­carboxaldehyde and (*S*)-(−)-1-phenyl­ethyl­amine, and coordination to HgCl_2_ afforded complex (I), [HgCl_2_ *L* ^1^]. The monoclinic unit cell contains four mol­ecules per asymmetric unit (Fig. 1 ▸), each one displaying a slightly different conformation for the ligand. The imine bond is coplanar with the pyridine ring in all independent mol­ecules, favoring the coordination of both N donors of *L* ^1^ to the metal. However, the phenyl ring has a degree of free rotation, generating four conformers: the observed dihedral angles between the pyridine and phenyl rings in complexes built on Hg1, Hg2, Hg3 and Hg4, are 71.1 (6), 78.0 (5), 82.3 (4) and 86.3 (6)°, respectively. These angles thus span a quite broad range of *ca* 15°, which could account for the *Z*′ = 4 character of the crystal.

Regarding the coordination geometry, the four complexes present an arrangement inter­mediate between tetra­hedral and disphenoidal. The N—Hg—N bite angles formed by the Schiff base range from 69.7 (5) to 71.3 (5)°, confirming the rigid character of this part of *L* ^1^. In contrast, Cl—Hg—Cl angles are found in a larger range, from 116.0 (2) to 126.78 (17)° (Table 1 ▸). The coordination is however far from the idealized *C*_2v_-disphenoidal or *C*_3v_-trigonal pyramid arrangements.

**Figure 2:**
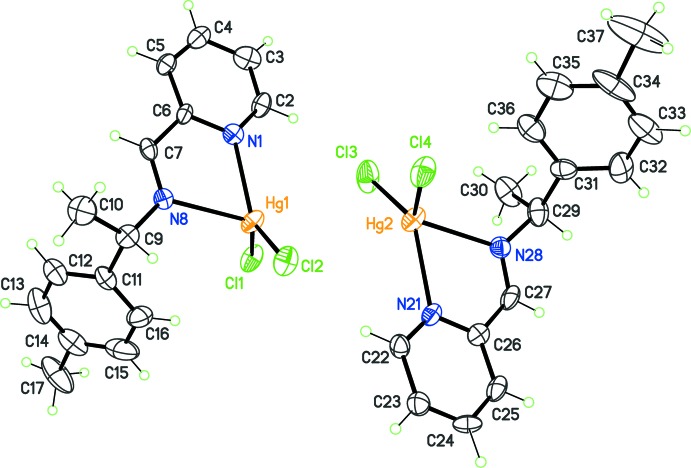
The asymmetric unit for complex (II), with displacement ellipsoids at the 30% probability level.

Ligand *L* ^2^ was obtained using (*S*)-(−)-1-(4-methyl­phen­yl)ethyl­amine for the Schiff condensation, and complex (II), [HgCl_2_ *L* ^2^] crystallized in the triclinic system, with two independent mol­ecules in the asymmetric unit (Fig. 2 ▸). Although the relative position of these mol­ecules emulates a non-crystallographic inversion centre, the structure was refined in space group *P*1 on the basis of the chiral nature of (II). The correctness of this choice was confirmed by the refinement of the Flack parameter (see *Refinement* section). Geometric features related to the conformation for *L* ^2^ and to its coordination geometry are compiled in Table 1 ▸, for comparison purposes. As expected, only small differences between (I) and (II) are observed. The most significant difference is for the bent conformation of the ligand, since *L* ^1^ seems to be more flexible than *L* ^2^. This difference could be sufficient to produce a symmetry reduction from *P*2_1_ to *P*1, accompanied by the halving of independent conformers in the crystals, from *Z*′ = 4 to *Z*′ = 2.

**Figure 3:**
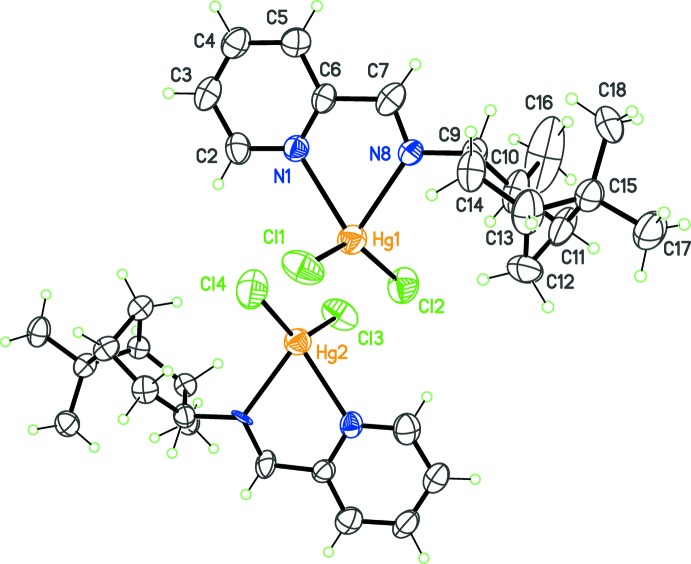
The asymmetric unit for complex (III), with displacement ellipsoids at the 30% probability level. The labels for C and N atoms in mol­ecule Hg2 are as in mol­ecule Hg1, but increased by 20.

The third imine, *L* ^3^, was obtained by condensation between 2-pyridine­carboxaldehyde and (1*S*,2*S*,3*S*,5*R*)-(+)-isopino­campheyl­amine. The complex formed upon coordination to HgCl_2_, (III), crystallizes with two mol­ecules in the asymmetric unit (Fig. 3 ▸), which have very similar conformations: the r.m.s.d. for a fit between the independent mol­ecules is 0.47 Å (Macrae *et al.*, 2008 ▸). As for (II), the independent mol­ecules are related by a non-crystallographic inversion centre, at least until chiral centres are considered. The bite angle formed by *L* ^3^ is comparable to that formed by *L* ^1^ or *L* ^2^ (Table 1 ▸). However, in the case of (III), the Cl—Hg—Cl angles are larger and, as a consequence, the tetra­hedral coordination geometry in that case is more distorted towards the *C*_2v_-disphenoidal geometry, compared to (I) and (II). No robust correlations between N—Hg—N and Cl—Hg—Cl angles were found after mining the CSD for tetra­coordinated Hg^II^ complexes, making a rationalization on distortion trends in these complexes difficult to draw.

## Supra­molecular features   

**Figure 4:**
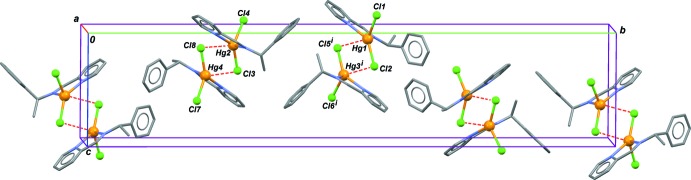
A part of the crystal structure of (I), emphasizing the aggregation of complexes in form of dimers. Dashed red bonds are non-covalent Hg⋯Cl inter­molecular contacts forming dimeric species. H atoms have been omitted. [Symmetry code: (i) *x* − 1, *y*, *z*.]

The most preeminent feature in the crystal structures of (I)–(III) is related to their multi-*Z*′ character. Within and beyond asymmetric units, inter­molecular Hg⋯Cl contacts are observed, which could be inter­preted as a pattern of dimerization, to form complexes of formula [Hg_2_ *L_2_*(*μ*-Cl)_2_Cl_2_]. For (I), mol­ecules based on Hg1 and Hg3 give contacts Hg1⋯Cl5^i^ = 3.172 (6) Å and Hg3^i^⋯Cl2 = 3.258 (5) Å (symmetry code: (i) −1 + *x*, *y*, *z*; sum of van der Waals radii: 3.3 Å; Bondi, 1964 ▸). In the asymmetric unit, mol­ecules based on Hg2 and Hg4 aggregate in a similar manner, with separations Hg2⋯Cl8^ii^ = 3.189 (5) Å and Cl3⋯Hg4^ii^ = 3.021 (6) Å [symmetry code: (ii) 1 − *x*, −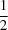 + *y*, 1 − *z*). The resulting asymmetric dimers are arranged in the crystal as depicted in Fig. 4 ▸.

The same dimerization tendency is observed for *Z*′ = 2 structures: in the crystal structure of (II), the asymmetric (*μ*-Cl)_2_ double bridge is characterized by separations Hg1⋯Cl3 = 3.089 (9) Å and Hg2⋯Cl2 = 3.211 (8) Å. In the crystal structure of (III), the asymmetry of the bridge is more pronounced, with separations Hg1⋯Cl4 = 3.395 (8) Å and Hg2⋯Cl2 = 3.564 (9) Å, longer than the sum of van der Waals radii for Hg and Cl.

The point of inter­est is that in all cases, the dimeric species are formed through a non-crystallographic inversion centre, if chiral centres in ligands *L* ^1–3^ are ignored. Since the chiral nature of the complexes forces them to crystallize in a Sohncke space group, the stabilization of the crystal structures through the formation of such pseudo-centrosymmetric dimers is possible only if *Z*′ > 1, as observed. On the other hand, it appears that the coordination geometry in the reported complexes is far enough from a disphenoidal geometry in order to promote dimerization. Indeed, the idealized *C*_2v_-disphenoidal coordination would prevent the formation of the (*μ*-Cl)_2_ bridge, since in that case the metal⋯metal separation would become too short.

## Database survey   

The crystal structures of *L* ^1–3^ remain unknown, presumably because these compounds are obtained as oils at room temperature. However, *L* ^1^ has been widely used as a ligand for coordination chemistry. The current release of the CSD (Version 5.36 with all updates; Groom & Allen, 2014 ▸) reports complexes with numerous transition metals, for example Mn^II^, Zn^II^, Ni^II^, Co^II^ and Co^III^ (Howson *et al.*, 2011 ▸), Cu^II^ (Min *et al.*, 2010 ▸), Pd^II^ (Mishnev *et al.*, 2000 ▸), and Rh^III^ (Carmona *et al.*, 1999 ▸). Nevertheless, no crystal structures have been deposited for Hg^II^ complexes. An Hg^II^ complex bearing a non-chiral Schiff base close to *L* ^1^ has been published (Kim & Kang, 2010 ▸). There are no structures including ligands *L* ^2^ or *L* ^3^ deposited in the CSD.

## Biological activity   

The anti­microbial activity of the complexes (I)–(III) was evaluated against Gram positive (*Staphylococcus aureus*) and Gram negative (*E. coli*, *Pseudomonas aeruginosa*) bacteria, and yeast (*Candida albicans*). All complexes were found to possess noteworthy anti­microbial activity (see supporting information). Among the compounds analyzed, (I) and (III) show high anti­microbial activity against all strains assessed. In general, all complexes tested displayed anti­fungal activity against the strains of *C. albicans*.

## Synthesis and crystallization   

**Caution!!** Any mercury compound poses potential health risks, and appropriate safety precautions along with disposal procedures must be taken in handling the complexes here reported. HgCl_2_ sublimes to emit highly poisonous fumes, and must be handled only by trained persons, under appropriate conditions.

**Synthesis of ligands**. Compounds *L* ^1–3^ were obtained by direct reaction between equimolar amounts of 2-pyridine­carboxaldehyde (1.6 g., 15 mmol) and the suitable optically active amine, (*S*)-(−)-1-phenyl­ethyl­amine (affording *L* ^1^, yield: 95%), (*S*)-(−)-1-(4-methyl­phen­yl)ethyl­amine (affording *L* ^2^, yield: 93%), or (1*S*,2*S*,3*S*,5*R*)-(+)-isopinocampheyl­amine (affording *L* ^3^, yield: 90%), under solvent-free conditions. The products, obtained as light-yellow oils, were characterized by spectroscopic techniques (see supporting information) and were used without further purification.

**Synthesis of complexes**. A solution of the chiral imine *L* ^1–3^ (0.35 mmol) in methanol (20 ml) was treated with HgCl_2_ (0.1 g, 0.35 mmol) with stirring at room temperature for 1 h. The solid obtained was filtered out and dried *in vacuo*, and then dissolved in di­chloro­methane. The resulting solution was slowly evaporated in a non-controlled atmosphere, and after a few days, colourless crystals of complexes (I)–(III) were collected, with yields of 81, 75, and 77%, respectively. Spectroscopic data are available from the supporting information.

## Refinement   

Crystal data, data collection and structure refinement details are summarized in Table 2 ▸. In the case of the triclinic crystal (II), the refined model contains a pseudo-inversion centre, at a confidence level of 95%. However, Wilson statistics, 〈|*E* ^2^ − 1|〉 = 0.726, point to the space group *P*1. This is confirmed by the optical activity measured for (II), and the convergence of the Flack parameter to the expected value. For (II), diffraction data for two crystals from different synthesis were collected, giving the same space group and final model. The best data set has been retained. However, due to strong correlations between parameters of *p*-tolyl groups in the independent mol­ecules, these groups were restrained to have the same geometry, with effective standard deviations of 0.02 and 0.04 Å for the 1,2- and 1,3-distances, respectively (*SAME* command in *SHELXL*; Sheldrick, 2015 ▸). In all structures, H atoms were placed in idealized positions and refined in the riding approximation, with C—H distances constrained to 0.93 (aromatic CH), 0.96 (methyl CH_3_), 0.97 (methyl­ene CH_2_) or 0.98 Å (methine CH). Isotropic displacement parameters for H atoms were calculated as *U*_iso_(H) = *xU*_eq_(carrier C), with *x* = 1.5 (methyl groups) or 1.2 (other H atoms).

## Supplementary Material

Crystal structure: contains datablock(s) I, II, III, global. DOI: 10.1107/S2056989015020368/rz5175sup1.cif

Structure factors: contains datablock(s) I. DOI: 10.1107/S2056989015020368/rz5175Isup2.hkl

Structure factors: contains datablock(s) II. DOI: 10.1107/S2056989015020368/rz5175IIsup3.hkl

Structure factors: contains datablock(s) III. DOI: 10.1107/S2056989015020368/rz5175IIIsup4.hkl

Supporting information file. DOI: 10.1107/S2056989015020368/rz5175sup5.pdf

CCDC references: 1433633, 1433632, 1433631

Additional supporting information:  crystallographic information; 3D view; checkCIF report

## Figures and Tables

**Table 1:** Comparison of key conformation parameters (°) for compounds (I), (II) and (III)

Compound/Mol­ecule	δ_py—Ph_ *^*a*^*	Bite angle*^*b*^*	Cl—Hg—Cl
(I)/Hg1	71.1 (6)	69.7 (5)	122.39 (19)
(I)/Hg2	78.0 (5)	70.4 (5)	117.1 (2)
(I)/Hg3	82.3 (4)	71.3 (5)	116.0 (2)
(I)/Hg4	86.3 (6)	69.9 (5)	126.78 (17)
			
(II)/Hg1	78.5 (7)	71.3 (7)	129.6 (2)
(II)/Hg2	78.2 (7)	70.1 (7)	121.7 (3)
			
(III)/Hg1	-	69.3 (7)	138.3 (3)
(III)/Hg2	-	70.3 (7)	132.1 (4)

Notes: (*a*) dihedral angle between aromatic rings in the ligand *L*; (*b*) N—Hg—N angle.

**Table 2:** Experimental details

	(I)	(II)	(III)
Crystal data			
Chemical formula	[HgCl_2_(C_14_H_14_N_2_)]	[HgCl_2_(C_15_H_16_N_2_)]	[HgCl_2_(C_16_H_22_N_2_)]
*M*_r_	481.76	495.79	513.84
Crystal system, space group	Monoclinic, *P*2_1_	Triclinic, *P*1	Monoclinic, *P*2_1_
Temperature (K)	298	294	298
*a*, *b*, *c* (Å)	7.5335 (12), 43.246 (6), 9.3069 (11)	7.6194 (3), 9.2982 (4), 12.2341 (8)	10.216 (3), 7.392 (2), 23.352 (6)
α, β, γ (°)	90, 90.486 (15), 90	94.597 (4), 103.178 (4), 94.222 (3)	90, 97.459 (14), 90
*V* (Å^3^)	3032.0 (7)	837.43 (7)	1748.6 (8)
*Z*	8	2	4
Radiation type	Mo *K*α	Mo *K*α	Mo *K*α
μ (mm^−1^)	10.49	9.50	9.10
Crystal size (mm)	0.50 × 0.18 × 0.08	0.67 × 0.36 × 0.11	0.4 × 0.2 × 0.1

Data collection			
Diffractometer	Bruker P4	Agilent Xcalibur (Atlas, Gemini)	Bruker P4
Absorption correction	ψ scan (*XSCANS*; Fait, 1996 ▸)	Analytical (*CrysAlis PRO*; Agilent, 2013 ▸)	Part of the refinement model (Δ*F*) (Walker & Stuart, 1983 ▸)
*T*_min_, *T*_max_	0.205, 0.431	0.052, 0.467	0.075, 0.405
No. of measured, independent and observed [*I* > 2σ(*I*)] reflections	11312, 5884, 4961	17266, 6767, 5013	9195, 6573, 4910
*R*_int_	0.049	0.046	0.045
(sin θ/λ)_max_ (Å^−1^)	0.595	0.625	0.622

Refinement			
*R*[*F* ^2^ > 2σ(*F* ^2^)], *wR*(*F* ^2^), *S*	0.042, 0.101, 1.06	0.036, 0.059, 0.98	0.057, 0.166, 1.11
No. of reflections	5884	6767	6573
No. of parameters	689	365	386
No. of restraints	1	18	1
H-atom treatment	H-atom parameters constrained	H-atom parameters constrained	H-atom parameters constrained
Δρ_max_, Δρ_min_ (e Å^−3^)	0.99, −2.17	1.07, −1.06	1.84, −1.76
Absolute structure	Classical Flack method preferred over Parsons because s.u. lower; 497 Friedel pairs measured	Flack *x* determined using 1903 quotients [(*I* ^+^)−(*I* ^−^)]/[(*I* ^+^)+(*I* ^−^)] (Parsons *et al.*, 2013 ▸).	Flack *x* determined using 1701 quotients [(*I* ^+^)−(*I* ^−^)]/[(*I* ^+^)+(*I* ^−^)] (Parsons *et al.*, 2013 ▸)
Absolute structure parameter	−0.011 (10)	−0.006 (12)	−0.05 (2)

Computer programs: *XSCANS* (Fait, 1996 ▸), *CrysAlis PRO* (Agilent, 2013 ▸), *SHELXS2014/7* (Sheldrick, 2008 ▸), *SHELXL2014/7* (Sheldrick, 2015 ▸) and *Mercury* (Macrae *et al.*, 2008 ▸).

## supplementary crystallographic information

### (I) (S)-(+)-Dichlorido[1-phenyl-N-(pyridin-2-ylmethylidene)ethylamine-κ2N,N']mercury(II) Crystal data

**Table:**

[HgCl_2_(C_14_H_14_N_2_)]	*D*_x_ = 2.111 Mg m^−^^3^
*M**_r_* = 481.76	Melting point: 412 K
Monoclinic, *P*2_1_	Mo *K*α radiation, λ = 0.71073 Å
*a* = 7.5335 (12) Å	Cell parameters from 80 reflections
*b* = 43.246 (6) Å	θ = 4.7–12.4°
*c* = 9.3069 (11) Å	µ = 10.49 mm^−^^1^
β = 90.486 (15)°	*T* = 298 K
*V* = 3032.0 (7) Å^3^	Plate, colourless
*Z* = 8	0.50 × 0.18 × 0.08 mm
*F*(000) = 1808	

### (I) (S)-(+)-Dichlorido[1-phenyl-N-(pyridin-2-ylmethylidene)ethylamine-κ2N,N']mercury(II) Data collection

**Table:**

Bruker P4 diffractometer	4961 reflections with *I* > 2σ(*I*)
Radiation source: fine-focus sealed tube, FN4	*R*_int_ = 0.049
Graphite monochromator	θ_max_ = 25.0°, θ_min_ = 2.2°
ω scans	*h* = −8→8
Absorption correction: ψ scan (XSCANS; Fait, 1996)	*k* = −51→39
*T*_min_ = 0.205, *T*_max_ = 0.431	*l* = −11→11
11312 measured reflections	3 standard reflections every 97 reflections
5884 independent reflections	intensity decay: 1.5%

### (I) (S)-(+)-Dichlorido[1-phenyl-N-(pyridin-2-ylmethylidene)ethylamine-κ2N,N']mercury(II) Refinement

**Table:**

Refinement on *F*^2^	Secondary atom site location: difference Fourier map
Least-squares matrix: full	Hydrogen site location: inferred from neighbouring sites
*R*[*F*^2^ > 2σ(*F*^2^)] = 0.042	H-atom parameters constrained
*wR*(*F*^2^) = 0.101	*w* = 1/[σ^2^(*F*_o_^2^) + (0.0612*P*)^2^] where *P* = (*F*_o_^2^ + 2*F*_c_^2^)/3
*S* = 1.06	(Δ/σ)_max_ = 0.002
5884 reflections	Δρ_max_ = 0.99 e Å^−^^3^
689 parameters	Δρ_min_ = −2.17 e Å^−^^3^
1 restraint	Absolute structure: Classical Flack method preferred over Parsons because s.u. lower; 497 Friedel pairs measured.
0 constraints	Absolute structure parameter: −0.011 (10)
Primary atom site location: structure-invariant direct methods	

### (I) (S)-(+)-Dichlorido[1-phenyl-N-(pyridin-2-ylmethylidene)ethylamine-κ2N,N']mercury(II) Fractional atomic coordinates and isotropic or equivalent isotropic displacement parameters (Å^2^)

**Table:**

	*x*	*y*	*z*	*U*_iso_*/*U*_eq_	
Hg1	−0.13474 (9)	0.52723 (2)	0.04097 (7)	0.0519 (2)	
Cl1	−0.2879 (7)	0.54184 (12)	−0.1805 (5)	0.0573 (12)	
Cl2	−0.2514 (8)	0.53847 (12)	0.2722 (5)	0.0697 (15)	
N1	0.0433 (19)	0.4915 (3)	−0.0830 (13)	0.042 (3)	
C2	−0.025 (3)	0.4680 (4)	−0.1581 (17)	0.050 (4)	
H2A	−0.1451	0.4633	−0.1485	0.060*	
C3	0.078 (3)	0.4506 (4)	−0.2499 (18)	0.049 (4)	
H3A	0.0277	0.4346	−0.3031	0.059*	
C4	0.251 (3)	0.4572 (4)	−0.2613 (18)	0.052 (5)	
H4A	0.3237	0.4454	−0.3198	0.062*	
C5	0.318 (2)	0.4818 (4)	−0.1844 (17)	0.044 (4)	
H5A	0.4384	0.4867	−0.1904	0.052*	
C6	0.209 (2)	0.4993 (4)	−0.0988 (16)	0.041 (4)	
C7	0.273 (2)	0.5258 (4)	−0.0201 (18)	0.051 (4)	
H7A	0.3935	0.5301	−0.0191	0.061*	
N8	0.1696 (18)	0.5433 (3)	0.0470 (12)	0.038 (3)	
C9	0.241 (3)	0.5717 (4)	0.126 (2)	0.055 (5)	
H9A	0.2722	0.5659	0.2245	0.066*	
C10	0.403 (3)	0.5860 (5)	0.056 (3)	0.084 (7)	
H10A	0.4432	0.6033	0.1126	0.126*	
H10B	0.3731	0.5929	−0.0389	0.126*	
H10C	0.4964	0.5709	0.0513	0.126*	
C11	0.096 (2)	0.5955 (4)	0.1296 (18)	0.044 (4)	
C12	0.047 (3)	0.6080 (5)	0.264 (2)	0.070 (6)	
H12A	0.0975	0.6000	0.3474	0.084*	
C13	−0.073 (4)	0.6317 (5)	0.274 (3)	0.094 (9)	
H13A	−0.0998	0.6404	0.3625	0.113*	
C14	−0.154 (3)	0.6426 (5)	0.150 (4)	0.097 (10)	
H14A	−0.2390	0.6581	0.1547	0.116*	
C15	−0.109 (4)	0.6305 (5)	0.020 (3)	0.100 (10)	
H15A	−0.1620	0.6383	−0.0631	0.120*	
C16	0.015 (3)	0.6067 (5)	0.008 (2)	0.069 (6)	
H16A	0.0432	0.5986	−0.0812	0.083*	
Hg2	0.00753 (9)	0.27508 (2)	0.10580 (7)	0.0529 (2)	
Cl3	0.1318 (8)	0.28498 (13)	0.3397 (6)	0.0773 (17)	
Cl4	0.1643 (7)	0.29633 (14)	−0.0993 (6)	0.0682 (14)	
N21	−0.1699 (18)	0.2386 (3)	−0.0054 (14)	0.041 (3)	
C22	−0.112 (3)	0.2127 (4)	−0.070 (2)	0.055 (5)	
H22A	0.0060	0.2068	−0.0571	0.066*	
C23	−0.220 (3)	0.1946 (4)	−0.155 (2)	0.062 (5)	
H23A	−0.1759	0.1765	−0.1943	0.075*	
C24	−0.391 (3)	0.2028 (4)	−0.182 (2)	0.060 (5)	
H24A	−0.4638	0.1914	−0.2442	0.072*	
C25	−0.453 (2)	0.2298 (4)	−0.1107 (16)	0.044 (4)	
H25A	−0.5705	0.2361	−0.1222	0.053*	
C26	−0.340 (2)	0.2464 (3)	−0.0251 (17)	0.040 (4)	
C27	−0.402 (2)	0.2748 (4)	0.0464 (17)	0.043 (4)	
H27A	−0.5233	0.2791	0.0490	0.052*	
N28	−0.2969 (18)	0.2928 (3)	0.1024 (13)	0.036 (3)	
C29	−0.376 (3)	0.3222 (4)	0.1642 (19)	0.054 (5)	
H29A	−0.5021	0.3187	0.1834	0.065*	
C30	−0.283 (3)	0.3298 (4)	0.3019 (18)	0.064 (6)	
H30A	−0.2727	0.3114	0.3592	0.095*	
H30B	−0.3507	0.3450	0.3533	0.095*	
H30C	−0.1674	0.3378	0.2821	0.095*	
C31	−0.359 (3)	0.3477 (3)	0.0512 (18)	0.048 (4)	
C32	−0.192 (3)	0.3576 (4)	0.012 (2)	0.063 (5)	
H32A	−0.0910	0.3490	0.0530	0.075*	
C33	−0.180 (4)	0.3810 (5)	−0.094 (2)	0.082 (7)	
H33A	−0.0689	0.3878	−0.1239	0.098*	
C34	−0.328 (4)	0.3936 (5)	−0.151 (2)	0.070 (6)	
H34A	−0.3192	0.4092	−0.2192	0.084*	
C35	−0.491 (4)	0.3838 (5)	−0.109 (2)	0.087 (8)	
H35A	−0.5919	0.3930	−0.1479	0.105*	
C36	−0.507 (3)	0.3607 (4)	−0.012 (2)	0.061 (5)	
H36A	−0.6193	0.3535	0.0121	0.073*	
Hg3	0.50699 (9)	0.47731 (2)	0.33042 (7)	0.0527 (2)	
Cl5	0.6382 (8)	0.46702 (14)	0.1035 (5)	0.0777 (17)	
Cl6	0.6675 (7)	0.45758 (13)	0.5410 (5)	0.0650 (13)	
N41	0.3328 (19)	0.5139 (3)	0.4461 (14)	0.041 (3)	
C42	0.397 (3)	0.5383 (4)	0.5172 (19)	0.054 (5)	
H42A	0.5170	0.5431	0.5069	0.065*	
C43	0.296 (3)	0.5566 (4)	0.605 (2)	0.057 (5)	
H43A	0.3446	0.5734	0.6544	0.068*	
C44	0.122 (3)	0.5492 (4)	0.618 (2)	0.056 (5)	
H44A	0.0490	0.5608	0.6772	0.068*	
C45	0.054 (2)	0.5248 (4)	0.5436 (17)	0.050 (4)	
H45A	−0.0658	0.5200	0.5503	0.060*	
C46	0.1616 (19)	0.5076 (3)	0.4601 (14)	0.032 (3)	
C47	0.109 (3)	0.4794 (4)	0.382 (2)	0.057 (5)	
H47A	−0.0130	0.4766	0.3700	0.068*	
N48	0.2003 (18)	0.4594 (3)	0.3300 (15)	0.042 (3)	
C49	0.126 (2)	0.4316 (4)	0.2696 (16)	0.042 (4)	
H49A	−0.0006	0.4353	0.2516	0.051*	
C50	0.212 (3)	0.4232 (5)	0.1270 (19)	0.068 (6)	
H50A	0.1664	0.4364	0.0526	0.102*	
H50B	0.1855	0.4021	0.1039	0.102*	
H50C	0.3383	0.4258	0.1349	0.102*	
C51	0.143 (2)	0.4065 (4)	0.3805 (17)	0.045 (4)	
C52	0.306 (3)	0.3955 (5)	0.427 (2)	0.071 (6)	
H52A	0.4092	0.4036	0.3874	0.085*	
C53	0.319 (4)	0.3732 (5)	0.530 (2)	0.080 (7)	
H53A	0.4301	0.3661	0.5590	0.096*	
C54	0.172 (5)	0.3614 (5)	0.589 (2)	0.098 (10)	
H54A	0.1818	0.3463	0.6593	0.117*	
C55	0.011 (4)	0.3715 (6)	0.546 (2)	0.084 (7)	
H55A	−0.0908	0.3634	0.5880	0.101*	
C56	−0.005 (3)	0.3938 (4)	0.441 (2)	0.057 (5)	
H56A	−0.1171	0.4002	0.4111	0.069*	
Hg4	0.65551 (9)	0.72714 (2)	0.59963 (7)	0.05081 (19)	
Cl7	0.8089 (7)	0.70943 (12)	0.3893 (5)	0.0607 (12)	
Cl8	0.7367 (7)	0.71712 (11)	0.8472 (5)	0.0608 (12)	
N61	0.4716 (19)	0.7631 (3)	0.4763 (14)	0.042 (3)	
C62	0.528 (3)	0.7869 (4)	0.4014 (18)	0.050 (5)	
H62A	0.6472	0.7925	0.4090	0.060*	
C63	0.418 (3)	0.8041 (4)	0.312 (2)	0.057 (5)	
H63A	0.4616	0.8207	0.2594	0.068*	
C64	0.247 (3)	0.7960 (4)	0.3020 (16)	0.054 (5)	
H64A	0.1691	0.8071	0.2431	0.065*	
C65	0.186 (3)	0.7712 (4)	0.3806 (18)	0.051 (4)	
H65A	0.0671	0.7657	0.3770	0.061*	
C66	0.300 (2)	0.7547 (3)	0.4626 (15)	0.037 (4)	
C67	0.241 (2)	0.7276 (4)	0.5469 (17)	0.044 (4)	
H67A	0.1209	0.7225	0.5495	0.053*	
N68	0.3517 (17)	0.7116 (4)	0.6147 (15)	0.047 (4)	
C69	0.304 (2)	0.6831 (4)	0.693 (2)	0.058 (5)	
H69A	0.3358	0.6862	0.7942	0.069*	
C70	0.111 (3)	0.6737 (5)	0.687 (3)	0.091 (8)	
H70A	0.0380	0.6911	0.7120	0.136*	
H70B	0.0908	0.6572	0.7545	0.136*	
H70C	0.0809	0.6668	0.5922	0.136*	
C71	0.416 (3)	0.6568 (4)	0.638 (2)	0.053 (5)	
C72	0.539 (3)	0.6417 (4)	0.731 (2)	0.061 (5)	
H72A	0.5509	0.6478	0.8260	0.073*	
C73	0.637 (4)	0.6187 (5)	0.678 (3)	0.085 (8)	
H73A	0.7127	0.6080	0.7398	0.102*	
C74	0.629 (4)	0.6104 (5)	0.538 (3)	0.089 (8)	
H74A	0.7058	0.5952	0.5044	0.106*	
C75	0.514 (4)	0.6235 (6)	0.449 (3)	0.087 (8)	
H75A	0.5041	0.6165	0.3552	0.105*	
C76	0.408 (3)	0.6482 (5)	0.497 (2)	0.074 (6)	
H76A	0.3323	0.6584	0.4336	0.089*	

### (I) (S)-(+)-Dichlorido[1-phenyl-N-(pyridin-2-ylmethylidene)ethylamine-κ2N,N']mercury(II) Atomic displacement parameters (Å^2^)

**Table:**

	*U*^11^	*U*^22^	*U*^33^	*U*^12^	*U*^13^	*U*^23^
Hg1	0.0462 (4)	0.0696 (4)	0.0401 (3)	0.0122 (4)	0.0074 (3)	−0.0021 (3)
Cl1	0.051 (3)	0.072 (3)	0.049 (2)	0.014 (2)	−0.001 (2)	0.006 (2)
Cl2	0.080 (4)	0.081 (3)	0.049 (3)	−0.017 (3)	0.026 (3)	−0.013 (2)
N1	0.042 (9)	0.058 (8)	0.026 (6)	0.022 (7)	0.001 (6)	0.007 (6)
C2	0.044 (11)	0.060 (12)	0.046 (10)	−0.003 (9)	0.008 (8)	−0.006 (8)
C3	0.052 (13)	0.048 (9)	0.047 (9)	0.005 (9)	0.002 (8)	−0.006 (7)
C4	0.064 (14)	0.043 (9)	0.048 (10)	0.028 (9)	0.019 (9)	−0.014 (8)
C5	0.032 (9)	0.050 (10)	0.049 (9)	0.004 (8)	0.012 (7)	−0.001 (8)
C6	0.040 (11)	0.047 (9)	0.037 (8)	0.010 (8)	0.016 (7)	0.000 (7)
C7	0.052 (11)	0.042 (9)	0.058 (10)	0.003 (9)	0.015 (9)	0.012 (9)
N8	0.044 (8)	0.044 (7)	0.025 (6)	0.012 (6)	−0.010 (6)	−0.017 (5)
C9	0.068 (14)	0.030 (8)	0.066 (12)	0.006 (8)	−0.001 (10)	−0.017 (8)
C10	0.050 (14)	0.085 (15)	0.12 (2)	0.008 (12)	0.003 (14)	−0.033 (14)
C11	0.029 (9)	0.055 (10)	0.048 (10)	−0.002 (8)	0.007 (8)	−0.009 (8)
C12	0.075 (16)	0.066 (12)	0.069 (13)	−0.001 (11)	0.017 (12)	−0.012 (10)
C13	0.11 (2)	0.050 (13)	0.12 (2)	−0.031 (14)	0.034 (19)	−0.037 (14)
C14	0.055 (16)	0.063 (15)	0.17 (3)	−0.021 (12)	0.042 (18)	−0.060 (19)
C15	0.13 (2)	0.055 (12)	0.12 (2)	0.021 (14)	−0.071 (19)	−0.015 (13)
C16	0.074 (15)	0.075 (13)	0.058 (12)	0.032 (12)	−0.021 (11)	−0.009 (10)
Hg2	0.0438 (4)	0.0721 (5)	0.0427 (4)	−0.0078 (4)	−0.0053 (3)	0.0016 (3)
Cl3	0.087 (4)	0.091 (4)	0.053 (3)	0.023 (3)	−0.032 (3)	−0.015 (2)
Cl4	0.046 (3)	0.100 (4)	0.059 (3)	−0.006 (3)	−0.001 (2)	0.028 (3)
N21	0.028 (8)	0.053 (8)	0.042 (8)	0.002 (6)	−0.006 (6)	0.002 (6)
C22	0.040 (11)	0.062 (11)	0.062 (11)	0.011 (9)	−0.014 (9)	−0.006 (9)
C23	0.068 (15)	0.054 (11)	0.066 (12)	0.016 (10)	0.007 (11)	−0.013 (9)
C24	0.041 (12)	0.075 (12)	0.064 (12)	−0.004 (10)	−0.029 (10)	−0.025 (10)
C25	0.033 (9)	0.054 (10)	0.045 (9)	0.004 (8)	−0.010 (7)	0.000 (8)
C26	0.027 (9)	0.043 (8)	0.051 (9)	−0.004 (7)	0.001 (8)	−0.001 (7)
C27	0.031 (9)	0.045 (8)	0.055 (9)	−0.002 (8)	0.007 (8)	0.008 (8)
N28	0.036 (8)	0.043 (7)	0.030 (6)	0.006 (6)	0.001 (6)	0.008 (5)
C29	0.051 (12)	0.052 (10)	0.059 (11)	0.013 (9)	0.025 (9)	0.002 (8)
C30	0.098 (17)	0.055 (10)	0.038 (9)	0.033 (11)	0.013 (10)	0.003 (8)
C31	0.066 (13)	0.034 (8)	0.044 (9)	0.004 (8)	0.006 (9)	−0.005 (7)
C32	0.055 (13)	0.067 (12)	0.066 (12)	−0.002 (10)	−0.013 (11)	0.023 (10)
C33	0.11 (2)	0.061 (13)	0.076 (15)	−0.036 (14)	0.003 (15)	0.011 (11)
C34	0.11 (2)	0.058 (12)	0.039 (10)	0.006 (13)	0.001 (12)	0.013 (9)
C35	0.13 (2)	0.070 (14)	0.064 (14)	0.034 (15)	−0.011 (15)	0.009 (11)
C36	0.071 (14)	0.052 (10)	0.058 (11)	0.013 (10)	−0.016 (10)	−0.010 (9)
Hg3	0.0446 (4)	0.0722 (4)	0.0413 (3)	0.0082 (4)	0.0055 (3)	0.0004 (3)
Cl5	0.085 (4)	0.094 (4)	0.054 (3)	−0.022 (3)	0.027 (3)	−0.018 (3)
Cl6	0.047 (3)	0.094 (3)	0.054 (3)	0.008 (3)	−0.003 (2)	0.022 (3)
N41	0.050 (10)	0.039 (7)	0.035 (7)	−0.002 (6)	0.006 (6)	0.001 (6)
C42	0.039 (11)	0.075 (12)	0.048 (10)	−0.002 (9)	0.005 (9)	0.003 (9)
C43	0.058 (14)	0.064 (11)	0.050 (10)	−0.010 (10)	0.023 (9)	−0.011 (9)
C44	0.063 (14)	0.049 (10)	0.058 (11)	0.027 (10)	0.018 (10)	0.010 (9)
C45	0.038 (10)	0.063 (11)	0.049 (9)	0.018 (9)	0.011 (8)	−0.004 (9)
C46	0.019 (8)	0.050 (9)	0.028 (7)	−0.001 (7)	0.002 (6)	0.009 (6)
C47	0.053 (12)	0.042 (9)	0.074 (12)	−0.002 (9)	−0.031 (10)	0.020 (9)
N48	0.033 (8)	0.036 (7)	0.056 (8)	0.005 (6)	−0.004 (7)	0.005 (6)
C49	0.031 (9)	0.058 (10)	0.037 (8)	0.001 (7)	−0.014 (7)	0.007 (7)
C50	0.087 (16)	0.072 (12)	0.045 (10)	−0.016 (12)	−0.013 (10)	−0.005 (9)
C51	0.046 (11)	0.050 (9)	0.039 (9)	−0.012 (8)	−0.002 (8)	−0.009 (7)
C52	0.071 (15)	0.068 (12)	0.073 (14)	0.014 (11)	−0.007 (12)	0.013 (11)
C53	0.10 (2)	0.068 (14)	0.071 (14)	0.018 (13)	−0.010 (14)	0.022 (11)
C54	0.20 (4)	0.042 (11)	0.051 (12)	−0.004 (17)	−0.005 (18)	0.011 (9)
C55	0.11 (2)	0.084 (16)	0.061 (14)	−0.024 (15)	0.033 (14)	−0.002 (12)
C56	0.063 (13)	0.042 (9)	0.066 (12)	−0.024 (9)	0.004 (10)	0.000 (9)
Hg4	0.0400 (4)	0.0726 (4)	0.0397 (3)	0.0087 (4)	−0.0042 (3)	−0.0015 (3)
Cl7	0.051 (3)	0.077 (3)	0.054 (3)	0.008 (2)	0.005 (2)	−0.014 (2)
Cl8	0.059 (3)	0.082 (3)	0.041 (2)	0.002 (2)	−0.009 (2)	0.001 (2)
N61	0.054 (9)	0.031 (6)	0.042 (7)	0.010 (6)	−0.010 (7)	−0.007 (6)
C62	0.047 (11)	0.059 (11)	0.044 (10)	−0.007 (9)	−0.021 (9)	0.011 (8)
C63	0.062 (14)	0.060 (11)	0.048 (10)	−0.012 (10)	0.001 (10)	0.003 (8)
C64	0.075 (15)	0.065 (11)	0.023 (8)	0.016 (11)	−0.004 (9)	−0.011 (7)
C65	0.051 (11)	0.051 (10)	0.050 (10)	0.015 (9)	0.002 (8)	−0.005 (8)
C66	0.044 (10)	0.041 (8)	0.025 (7)	−0.014 (7)	−0.004 (7)	−0.015 (6)
C67	0.030 (9)	0.041 (8)	0.060 (10)	−0.002 (8)	0.004 (8)	0.002 (8)
N68	0.011 (7)	0.081 (10)	0.049 (8)	0.003 (7)	0.005 (6)	−0.009 (7)
C69	0.037 (11)	0.076 (12)	0.060 (11)	0.009 (9)	0.009 (9)	0.019 (10)
C70	0.076 (17)	0.075 (14)	0.12 (2)	0.014 (12)	0.034 (15)	0.045 (14)
C71	0.051 (12)	0.055 (10)	0.053 (11)	−0.013 (9)	0.017 (9)	0.006 (8)
C72	0.053 (12)	0.066 (12)	0.062 (11)	0.018 (10)	−0.031 (10)	0.010 (9)
C73	0.12 (2)	0.049 (11)	0.088 (17)	0.027 (13)	−0.035 (16)	−0.001 (11)
C74	0.09 (2)	0.061 (14)	0.11 (2)	−0.007 (13)	0.033 (17)	−0.014 (14)
C75	0.11 (2)	0.089 (17)	0.065 (14)	−0.044 (16)	0.010 (15)	−0.020 (13)
C76	0.101 (19)	0.077 (14)	0.044 (11)	−0.013 (13)	0.008 (12)	0.021 (10)

### (I) (S)-(+)-Dichlorido[1-phenyl-N-(pyridin-2-ylmethylidene)ethylamine-κ2N,N']mercury(II) Geometric parameters (Å, º)

**Table:**

Hg1—N1	2.356 (13)	Hg3—N41	2.327 (13)
Hg1—Cl2	2.382 (5)	Hg3—Cl5	2.381 (5)
Hg1—N8	2.396 (14)	Hg3—N48	2.437 (14)
Hg1—Cl1	2.437 (4)	Hg3—Cl6	2.448 (5)
N1—C6	1.30 (2)	N41—C46	1.327 (19)
N1—C2	1.33 (2)	N41—C42	1.33 (2)
C2—C3	1.38 (2)	C42—C43	1.38 (3)
C2—H2A	0.9300	C42—H42A	0.9300
C3—C4	1.34 (3)	C43—C44	1.35 (3)
C3—H3A	0.9300	C43—H43A	0.9300
C4—C5	1.38 (2)	C44—C45	1.36 (3)
C4—H4A	0.9300	C44—H44A	0.9300
C5—C6	1.37 (2)	C45—C46	1.35 (2)
C5—H5A	0.9300	C45—H45A	0.9300
C6—C7	1.44 (2)	C46—C47	1.47 (2)
C7—N8	1.25 (2)	C47—N48	1.21 (2)
C7—H7A	0.9300	C47—H47A	0.9300
N8—C9	1.53 (2)	N48—C49	1.44 (2)
C9—C11	1.50 (2)	C49—C51	1.50 (2)
C9—C10	1.52 (3)	C49—C50	1.53 (2)
C9—H9A	0.9800	C49—H49A	0.9800
C10—H10A	0.9600	C50—H50A	0.9600
C10—H10B	0.9600	C50—H50B	0.9600
C10—H10C	0.9600	C50—H50C	0.9600
C11—C16	1.37 (3)	C51—C56	1.37 (2)
C11—C12	1.41 (3)	C51—C52	1.38 (3)
C12—C13	1.37 (3)	C52—C53	1.36 (3)
C12—H12A	0.9300	C52—H52A	0.9300
C13—C14	1.38 (4)	C53—C54	1.34 (4)
C13—H13A	0.9300	C53—H53A	0.9300
C14—C15	1.36 (4)	C54—C55	1.35 (4)
C14—H14A	0.9300	C54—H54A	0.9300
C15—C16	1.39 (3)	C55—C56	1.38 (3)
C15—H15A	0.9300	C55—H55A	0.9300
C16—H16A	0.9300	C56—H56A	0.9300
Hg2—N21	2.307 (13)	Hg4—N61	2.373 (12)
Hg2—Cl3	2.401 (5)	Hg4—N68	2.390 (13)
Hg2—N28	2.417 (13)	Hg4—Cl7	2.406 (5)
Hg2—Cl4	2.433 (5)	Hg4—Cl8	2.418 (4)
N21—C26	1.34 (2)	N61—C62	1.32 (2)
N21—C22	1.35 (2)	N61—C66	1.35 (2)
C22—C23	1.38 (3)	C62—C63	1.39 (3)
C22—H22A	0.9300	C62—H62A	0.9300
C23—C24	1.36 (3)	C63—C64	1.34 (3)
C23—H23A	0.9300	C63—H63A	0.9300
C24—C25	1.43 (2)	C64—C65	1.38 (3)
C24—H24A	0.9300	C64—H64A	0.9300
C25—C26	1.37 (2)	C65—C66	1.35 (2)
C25—H25A	0.9300	C65—H65A	0.9300
C26—C27	1.47 (2)	C66—C67	1.48 (2)
C27—N28	1.225 (19)	C67—N68	1.25 (2)
C27—H27A	0.9300	C67—H67A	0.9300
N28—C29	1.52 (2)	N68—C69	1.48 (2)
C29—C30	1.49 (3)	C69—C70	1.51 (3)
C29—C31	1.53 (2)	C69—C71	1.51 (3)
C29—H29A	0.9800	C69—H69A	0.9800
C30—H30A	0.9600	C70—H70A	0.9600
C30—H30B	0.9600	C70—H70B	0.9600
C30—H30C	0.9600	C70—H70C	0.9600
C31—C36	1.38 (3)	C71—C76	1.36 (3)
C31—C32	1.38 (3)	C71—C72	1.42 (3)
C32—C33	1.42 (3)	C72—C73	1.34 (3)
C32—H32A	0.9300	C72—H72A	0.9300
C33—C34	1.35 (3)	C73—C74	1.35 (4)
C33—H33A	0.9300	C73—H73A	0.9300
C34—C35	1.35 (3)	C74—C75	1.32 (4)
C34—H34A	0.9300	C74—H74A	0.9300
C35—C36	1.36 (3)	C75—C76	1.41 (3)
C35—H35A	0.9300	C75—H75A	0.9300
C36—H36A	0.9300	C76—H76A	0.9300
			
N1—Hg1—Cl2	142.4 (3)	N41—Hg3—Cl5	141.0 (4)
N1—Hg1—N8	69.7 (5)	N41—Hg3—N48	71.3 (5)
Cl2—Hg1—N8	106.3 (3)	Cl5—Hg3—N48	109.9 (4)
N1—Hg1—Cl1	91.3 (3)	N41—Hg3—Cl6	98.2 (4)
Cl2—Hg1—Cl1	122.39 (19)	Cl5—Hg3—Cl6	116.0 (2)
N8—Hg1—Cl1	113.0 (3)	N48—Hg3—Cl6	110.6 (3)
C6—N1—C2	120.4 (15)	C46—N41—C42	117.9 (15)
C6—N1—Hg1	115.8 (11)	C46—N41—Hg3	117.1 (10)
C2—N1—Hg1	122.4 (12)	C42—N41—Hg3	124.2 (13)
N1—C2—C3	121.3 (18)	N41—C42—C43	123.2 (18)
N1—C2—H2A	119.3	N41—C42—H42A	118.4
C3—C2—H2A	119.3	C43—C42—H42A	118.4
C4—C3—C2	119.1 (16)	C44—C43—C42	117.2 (18)
C4—C3—H3A	120.5	C44—C43—H43A	121.4
C2—C3—H3A	120.5	C42—C43—H43A	121.4
C3—C4—C5	118.7 (15)	C43—C44—C45	120.0 (17)
C3—C4—H4A	120.7	C43—C44—H44A	120.0
C5—C4—H4A	120.7	C45—C44—H44A	120.0
C6—C5—C4	120.3 (17)	C46—C45—C44	119.8 (17)
C6—C5—H5A	119.9	C46—C45—H45A	120.1
C4—C5—H5A	119.9	C44—C45—H45A	120.1
N1—C6—C5	120.1 (15)	N41—C46—C45	121.9 (15)
N1—C6—C7	117.5 (15)	N41—C46—C47	112.5 (15)
C5—C6—C7	122.3 (16)	C45—C46—C47	125.5 (16)
N8—C7—C6	122.1 (17)	N48—C47—C46	129.4 (17)
N8—C7—H7A	119.0	N48—C47—H47A	115.3
C6—C7—H7A	119.0	C46—C47—H47A	115.3
C7—N8—C9	120.4 (15)	C47—N48—C49	122.1 (15)
C7—N8—Hg1	114.2 (11)	C47—N48—Hg3	108.5 (12)
C9—N8—Hg1	125.4 (11)	C49—N48—Hg3	129.2 (11)
C11—C9—C10	108.8 (15)	N48—C49—C51	107.8 (12)
C11—C9—N8	107.8 (15)	N48—C49—C50	111.9 (14)
C10—C9—N8	114.0 (15)	C51—C49—C50	113.0 (15)
C11—C9—H9A	108.7	N48—C49—H49A	108.0
C10—C9—H9A	108.7	C51—C49—H49A	108.0
N8—C9—H9A	108.7	C50—C49—H49A	108.0
C9—C10—H10A	109.5	C49—C50—H50A	109.5
C9—C10—H10B	109.5	C49—C50—H50B	109.5
H10A—C10—H10B	109.5	H50A—C50—H50B	109.5
C9—C10—H10C	109.5	C49—C50—H50C	109.5
H10A—C10—H10C	109.5	H50A—C50—H50C	109.5
H10B—C10—H10C	109.5	H50B—C50—H50C	109.5
C16—C11—C12	118.5 (18)	C56—C51—C52	117.2 (17)
C16—C11—C9	122.8 (16)	C56—C51—C49	120.7 (16)
C12—C11—C9	118.6 (17)	C52—C51—C49	122.1 (17)
C13—C12—C11	122 (2)	C53—C52—C51	121 (2)
C13—C12—H12A	119.2	C53—C52—H52A	119.3
C11—C12—H12A	119.2	C51—C52—H52A	119.3
C12—C13—C14	119 (2)	C54—C53—C52	120 (2)
C12—C13—H13A	120.6	C54—C53—H53A	119.9
C14—C13—H13A	120.6	C52—C53—H53A	119.9
C15—C14—C13	120 (3)	C53—C54—C55	120 (2)
C15—C14—H14A	120.0	C53—C54—H54A	119.9
C13—C14—H14A	120.0	C55—C54—H54A	119.9
C14—C15—C16	122 (2)	C54—C55—C56	120 (2)
C14—C15—H15A	119.2	C54—C55—H55A	119.8
C16—C15—H15A	119.2	C56—C55—H55A	119.8
C11—C16—C15	119 (2)	C51—C56—C55	121 (2)
C11—C16—H16A	120.3	C51—C56—H56A	119.7
C15—C16—H16A	120.3	C55—C56—H56A	119.7
N21—Hg2—Cl3	138.5 (4)	N61—Hg4—N68	69.9 (5)
N21—Hg2—N28	70.4 (5)	N61—Hg4—Cl7	95.6 (4)
Cl3—Hg2—N28	108.5 (3)	N68—Hg4—Cl7	115.1 (4)
N21—Hg2—Cl4	100.9 (4)	N61—Hg4—Cl8	136.1 (3)
Cl3—Hg2—Cl4	117.1 (2)	N68—Hg4—Cl8	97.4 (4)
N28—Hg2—Cl4	109.7 (3)	Cl7—Hg4—Cl8	126.78 (17)
C26—N21—C22	117.6 (14)	C62—N61—C66	118.4 (14)
C26—N21—Hg2	116.1 (10)	C62—N61—Hg4	125.3 (12)
C22—N21—Hg2	125.5 (12)	C66—N61—Hg4	115.1 (10)
N21—C22—C23	122.4 (18)	N61—C62—C63	122.7 (18)
N21—C22—H22A	118.8	N61—C62—H62A	118.7
C23—C22—H22A	118.8	C63—C62—H62A	118.7
C24—C23—C22	121.3 (18)	C64—C63—C62	118.4 (18)
C24—C23—H23A	119.4	C64—C63—H63A	120.8
C22—C23—H23A	119.4	C62—C63—H63A	120.8
C23—C24—C25	116.0 (15)	C63—C64—C65	119.2 (18)
C23—C24—H24A	122.0	C63—C64—H64A	120.4
C25—C24—H24A	122.0	C65—C64—H64A	120.4
C26—C25—C24	120.0 (15)	C66—C65—C64	120.1 (18)
C26—C25—H25A	120.0	C66—C65—H65A	119.9
C24—C25—H25A	120.0	C64—C65—H65A	119.9
N21—C26—C25	122.7 (15)	N61—C66—C65	121.1 (15)
N21—C26—C27	117.2 (14)	N61—C66—C67	117.3 (14)
C25—C26—C27	120.1 (15)	C65—C66—C67	121.5 (16)
N28—C27—C26	120.8 (15)	N68—C67—C66	120.0 (15)
N28—C27—H27A	119.6	N68—C67—H67A	120.0
C26—C27—H27A	119.6	C66—C67—H67A	120.0
C27—N28—C29	115.9 (15)	C67—N68—C69	123.0 (14)
C27—N28—Hg2	114.6 (11)	C67—N68—Hg4	116.8 (12)
C29—N28—Hg2	129.4 (11)	C69—N68—Hg4	120.0 (11)
C30—C29—N28	109.1 (14)	N68—C69—C70	116.5 (15)
C30—C29—C31	113.1 (16)	N68—C69—C71	108.7 (14)
N28—C29—C31	108.0 (13)	C70—C69—C71	109.2 (18)
C30—C29—H29A	108.9	N68—C69—H69A	107.4
N28—C29—H29A	108.9	C70—C69—H69A	107.4
C31—C29—H29A	108.9	C71—C69—H69A	107.4
C29—C30—H30A	109.5	C69—C70—H70A	109.5
C29—C30—H30B	109.5	C69—C70—H70B	109.5
H30A—C30—H30B	109.5	H70A—C70—H70B	109.5
C29—C30—H30C	109.5	C69—C70—H70C	109.5
H30A—C30—H30C	109.5	H70A—C70—H70C	109.5
H30B—C30—H30C	109.5	H70B—C70—H70C	109.5
C36—C31—C32	119.7 (17)	C76—C71—C72	119 (2)
C36—C31—C29	121.0 (18)	C76—C71—C69	120.7 (19)
C32—C31—C29	119.3 (17)	C72—C71—C69	120.1 (17)
C31—C32—C33	118 (2)	C73—C72—C71	118.6 (19)
C31—C32—H32A	120.8	C73—C72—H72A	120.7
C33—C32—H32A	120.8	C71—C72—H72A	120.7
C34—C33—C32	120 (2)	C72—C73—C74	122 (2)
C34—C33—H33A	120.0	C72—C73—H73A	118.9
C32—C33—H33A	120.0	C74—C73—H73A	118.9
C33—C34—C35	121 (2)	C75—C74—C73	121 (2)
C33—C34—H34A	119.7	C75—C74—H74A	119.6
C35—C34—H34A	119.7	C73—C74—H74A	119.6
C34—C35—C36	121 (2)	C74—C75—C76	120 (2)
C34—C35—H35A	119.7	C74—C75—H75A	120.0
C36—C35—H35A	119.7	C76—C75—H75A	120.0
C35—C36—C31	121 (2)	C71—C76—C75	119 (2)
C35—C36—H36A	119.7	C71—C76—H76A	120.4
C31—C36—H36A	119.7	C75—C76—H76A	120.4
			
C6—N1—C2—C3	3 (2)	C46—N41—C42—C43	1 (3)
Hg1—N1—C2—C3	168.5 (12)	Hg3—N41—C42—C43	−168.4 (14)
N1—C2—C3—C4	1 (3)	N41—C42—C43—C44	0 (3)
C2—C3—C4—C5	−2 (3)	C42—C43—C44—C45	−1 (3)
C3—C4—C5—C6	0 (3)	C43—C44—C45—C46	2 (3)
C2—N1—C6—C5	−5 (2)	C42—N41—C46—C45	0 (2)
Hg1—N1—C6—C5	−172.1 (12)	Hg3—N41—C46—C45	169.9 (12)
C2—N1—C6—C7	176.9 (14)	C42—N41—C46—C47	−177.4 (14)
Hg1—N1—C6—C7	10.0 (18)	Hg3—N41—C46—C47	−7.3 (16)
C4—C5—C6—N1	4 (2)	C44—C45—C46—N41	−1 (2)
C4—C5—C6—C7	−178.0 (15)	C44—C45—C46—C47	175.8 (15)
N1—C6—C7—N8	−9 (2)	N41—C46—C47—N48	14 (2)
C5—C6—C7—N8	173.3 (15)	C45—C46—C47—N48	−163.0 (18)
C6—C7—N8—C9	−178.2 (15)	C46—C47—N48—C49	173.3 (15)
C6—C7—N8—Hg1	2.7 (19)	C46—C47—N48—Hg3	−12 (2)
C7—N8—C9—C11	151.8 (15)	C47—N48—C49—C51	−99.1 (18)
Hg1—N8—C9—C11	−29.2 (19)	Hg3—N48—C49—C51	87.0 (16)
C7—N8—C9—C10	31 (2)	C47—N48—C49—C50	136.1 (17)
Hg1—N8—C9—C10	−150.1 (14)	Hg3—N48—C49—C50	−37.8 (18)
C10—C9—C11—C16	66 (2)	N48—C49—C51—C56	114.1 (16)
N8—C9—C11—C16	−58 (2)	C50—C49—C51—C56	−121.7 (17)
C10—C9—C11—C12	−110 (2)	N48—C49—C51—C52	−65 (2)
N8—C9—C11—C12	126.0 (18)	C50—C49—C51—C52	59 (2)
C16—C11—C12—C13	−2 (3)	C56—C51—C52—C53	−1 (3)
C9—C11—C12—C13	174.0 (19)	C49—C51—C52—C53	178.6 (19)
C11—C12—C13—C14	3 (4)	C51—C52—C53—C54	0 (3)
C12—C13—C14—C15	−2 (4)	C52—C53—C54—C55	0 (4)
C13—C14—C15—C16	1 (4)	C53—C54—C55—C56	0 (4)
C12—C11—C16—C15	1 (3)	C52—C51—C56—C55	1 (3)
C9—C11—C16—C15	−174.9 (19)	C49—C51—C56—C55	−177.8 (18)
C14—C15—C16—C11	−1 (4)	C54—C55—C56—C51	−1 (3)
C26—N21—C22—C23	0 (3)	C66—N61—C62—C63	1 (2)
Hg2—N21—C22—C23	−168.8 (16)	Hg4—N61—C62—C63	168.6 (14)
N21—C22—C23—C24	3 (3)	N61—C62—C63—C64	1 (3)
C22—C23—C24—C25	−4 (3)	C62—C63—C64—C65	0 (3)
C23—C24—C25—C26	2 (3)	C63—C64—C65—C66	−2 (2)
C22—N21—C26—C25	−2 (2)	C62—N61—C66—C65	−4 (2)
Hg2—N21—C26—C25	168.2 (13)	Hg4—N61—C66—C65	−172.1 (11)
C22—N21—C26—C27	180.0 (15)	C62—N61—C66—C67	179.4 (14)
Hg2—N21—C26—C27	−9.8 (18)	Hg4—N61—C66—C67	11.0 (16)
C24—C25—C26—N21	1 (3)	C64—C65—C66—N61	4 (2)
C24—C25—C26—C27	178.5 (16)	C64—C65—C66—C67	−179.2 (14)
N21—C26—C27—N28	11 (2)	N61—C66—C67—N68	−8 (2)
C25—C26—C27—N28	−167.2 (15)	C65—C66—C67—N68	175.4 (15)
C26—C27—N28—C29	176.2 (14)	C66—C67—N68—C69	−175.7 (15)
C26—C27—N28—Hg2	−5.9 (18)	C66—C67—N68—Hg4	0 (2)
C27—N28—C29—C30	140.9 (16)	C67—N68—C69—C70	0 (3)
Hg2—N28—C29—C30	−36.7 (19)	Hg4—N68—C69—C70	−175.4 (15)
C27—N28—C29—C31	−95.9 (18)	C67—N68—C69—C71	124.0 (18)
Hg2—N28—C29—C31	86.6 (17)	Hg4—N68—C69—C71	−51.6 (19)
C30—C29—C31—C36	−124.6 (18)	N68—C69—C71—C76	−61 (2)
N28—C29—C31—C36	114.6 (17)	C70—C69—C71—C76	67 (2)
C30—C29—C31—C32	56 (2)	N68—C69—C71—C72	116.3 (18)
N28—C29—C31—C32	−65 (2)	C70—C69—C71—C72	−116 (2)
C36—C31—C32—C33	0 (3)	C76—C71—C72—C73	−3 (3)
C29—C31—C32—C33	179.6 (17)	C69—C71—C72—C73	−180 (2)
C31—C32—C33—C34	1 (3)	C71—C72—C73—C74	3 (4)
C32—C33—C34—C35	−1 (3)	C72—C73—C74—C75	−5 (4)
C33—C34—C35—C36	−1 (4)	C73—C74—C75—C76	5 (4)
C34—C35—C36—C31	3 (3)	C72—C71—C76—C75	3 (3)
C32—C31—C36—C35	−2 (3)	C69—C71—C76—C75	−179.9 (18)
C29—C31—C36—C35	178.3 (17)	C74—C75—C76—C71	−4 (3)

### (II) (S)-(+)-Dichlorido[1-(4-methylphenyl)-N-(pyridin-2-ylmethylidene)ethylamine-κ2N,N']mercury(II) Crystal data

**Table:**

[HgCl_2_(C_15_H_16_N_2_)]	*F*(000) = 468
*M**_r_* = 495.79	*D*_x_ = 1.966 Mg m^−^^3^
Triclinic, *P*1	Melting point: 418 K
*a* = 7.6194 (3) Å	Mo *K*α radiation, λ = 0.71073 Å
*b* = 9.2982 (4) Å	Cell parameters from 4707 reflections
*c* = 12.2341 (8) Å	θ = 3.6–24.6°
α = 94.597 (4)°	µ = 9.50 mm^−^^1^
β = 103.178 (4)°	*T* = 294 K
γ = 94.222 (3)°	Plate, colourless
*V* = 837.43 (7) Å^3^	0.67 × 0.36 × 0.11 mm
*Z* = 2	

### (II) (S)-(+)-Dichlorido[1-(4-methylphenyl)-N-(pyridin-2-ylmethylidene)ethylamine-κ2N,N']mercury(II) Data collection

**Table:**

Agilent Xcalibur (Atlas, Gemini) diffractometer	6767 independent reflections
Radiation source: Enhance (Mo) X-ray Source	5013 reflections with *I* > 2σ(*I*)
Graphite monochromator	*R*_int_ = 0.046
Detector resolution: 10.5564 pixels mm^-1^	θ_max_ = 26.4°, θ_min_ = 2.9°
ω scans	*h* = −9→9
Absorption correction: analytical (CrysAlis PRO; Agilent, 2013)	*k* = −11→11
*T*_min_ = 0.052, *T*_max_ = 0.467	*l* = −15→15
17266 measured reflections	

### (II) (S)-(+)-Dichlorido[1-(4-methylphenyl)-N-(pyridin-2-ylmethylidene)ethylamine-κ2N,N']mercury(II) Refinement

**Table:**

Refinement on *F*^2^	Secondary atom site location: difference Fourier map
Least-squares matrix: full	Hydrogen site location: inferred from neighbouring sites
*R*[*F*^2^ > 2σ(*F*^2^)] = 0.036	H-atom parameters constrained
*wR*(*F*^2^) = 0.059	*w* = 1/[σ^2^(*F*_o_^2^) + (0.0149*P*)^2^] where *P* = (*F*_o_^2^ + 2*F*_c_^2^)/3
*S* = 0.98	(Δ/σ)_max_ = 0.001
6767 reflections	Δρ_max_ = 1.07 e Å^−^^3^
365 parameters	Δρ_min_ = −1.06 e Å^−^^3^
18 restraints	Absolute structure: Flack *x* determined using 1903 quotients [(*I*^+^)-(*I*^-^)]/[(*I*^+^)+(*I*^-^)] (Parsons *et al.*, 2013).
Primary atom site location: structure-invariant direct methods	Absolute structure parameter: −0.006 (12)

### (II) (S)-(+)-Dichlorido[1-(4-methylphenyl)-N-(pyridin-2-ylmethylidene)ethylamine-κ2N,N']mercury(II) Fractional atomic coordinates and isotropic or equivalent isotropic displacement parameters (Å^2^)

**Table:**

	*x*	*y*	*z*	*U*_iso_*/*U*_eq_	
Hg1	0.35646 (4)	0.24939 (4)	0.25613 (4)	0.0504 (4)	
Cl1	0.5356 (9)	0.0590 (7)	0.3269 (8)	0.062 (2)	
Cl2	0.4313 (9)	0.5077 (7)	0.2824 (7)	0.0612 (18)	
N1	0.124 (3)	0.1109 (19)	0.1251 (19)	0.041 (5)	
C2	0.141 (3)	0.027 (2)	0.037 (3)	0.051 (7)	
H2A	0.2547	0.0296	0.0209	0.061*	
C3	0.006 (4)	−0.065 (3)	−0.033 (3)	0.065 (8)	
H3A	0.0268	−0.1233	−0.0928	0.078*	
C4	−0.166 (3)	−0.067 (2)	−0.008 (2)	0.056 (7)	
H4A	−0.2627	−0.1252	−0.0552	0.067*	
C5	−0.191 (3)	0.014 (2)	0.082 (2)	0.044 (6)	
H5A	−0.3035	0.0104	0.0997	0.053*	
C6	−0.043 (3)	0.106 (2)	0.148 (2)	0.037 (6)	
C7	−0.062 (3)	0.196 (2)	0.251 (2)	0.039 (5)	
H7A	−0.1753	0.2042	0.2659	0.047*	
N8	0.080 (3)	0.2634 (19)	0.3184 (19)	0.038 (5)	
C9	0.064 (3)	0.352 (2)	0.419 (2)	0.049 (6)	
H9A	0.1455	0.4401	0.4248	0.059*	
C10	−0.1166 (17)	0.4005 (14)	0.4218 (13)	0.070 (4)	
H10A	−0.1101	0.4544	0.4931	0.105*	
H10B	−0.2036	0.3175	0.4117	0.105*	
H10C	−0.1526	0.4610	0.3621	0.105*	
C11	0.152 (3)	0.266 (2)	0.5207 (19)	0.048 (6)	
C12	0.047 (3)	0.181 (3)	0.572 (2)	0.068 (8)	
H12A	−0.0780	0.1746	0.5482	0.082*	
C13	0.125 (4)	0.104 (3)	0.658 (2)	0.092 (11)	
H13A	0.0516	0.0541	0.6963	0.110*	
C14	0.307 (3)	0.099 (3)	0.689 (2)	0.079 (10)	
C15	0.415 (3)	0.184 (3)	0.639 (2)	0.098 (12)	
H15A	0.5403	0.1872	0.6621	0.118*	
C16	0.335 (2)	0.266 (3)	0.553 (2)	0.065 (8)	
H16A	0.4085	0.3198	0.5165	0.078*	
C17	0.396 (4)	0.005 (3)	0.780 (2)	0.125 (12)	
H17A	0.3041	−0.0583	0.7987	0.187*	
H17B	0.4591	0.0665	0.8463	0.187*	
H17C	0.4794	−0.0513	0.7520	0.187*	
Hg2	0.60575 (4)	0.54275 (4)	0.06949 (4)	0.0524 (4)	
Cl3	0.4965 (11)	0.2909 (7)	0.0418 (8)	0.084 (3)	
Cl4	0.4179 (9)	0.7262 (8)	−0.0077 (8)	0.069 (2)	
N21	0.833 (3)	0.680 (2)	0.209 (2)	0.043 (5)	
C22	0.795 (3)	0.762 (2)	0.294 (2)	0.045 (6)	
H22A	0.6781	0.7572	0.3062	0.054*	
C23	0.936 (3)	0.854 (3)	0.365 (3)	0.053 (7)	
H23A	0.9133	0.9085	0.4269	0.064*	
C24	1.100 (3)	0.866 (2)	0.346 (2)	0.050 (6)	
H24A	1.1913	0.9307	0.3920	0.060*	
C25	1.134 (3)	0.779 (3)	0.258 (2)	0.052 (7)	
H25A	1.2500	0.7822	0.2459	0.063*	
C26	0.996 (3)	0.690 (2)	0.189 (2)	0.041 (6)	
C27	1.022 (3)	0.609 (3)	0.090 (2)	0.050 (7)	
H27A	1.1391	0.6063	0.0804	0.060*	
N28	0.895 (3)	0.543 (2)	0.015 (2)	0.045 (5)	
C29	0.929 (3)	0.476 (2)	−0.094 (3)	0.061 (7)	
H29A	1.0584	0.4946	−0.0900	0.073*	
C30	0.884 (2)	0.3118 (14)	−0.1048 (13)	0.075 (4)	
H30A	0.9046	0.2699	−0.1746	0.113*	
H30B	0.7597	0.2901	−0.1034	0.113*	
H30C	0.9606	0.2723	−0.0429	0.113*	
C31	0.832 (3)	0.549 (2)	−0.190 (2)	0.055 (7)	
C32	0.922 (3)	0.632 (3)	−0.254 (2)	0.078 (9)	
H32A	1.0471	0.6344	−0.2410	0.094*	
C33	0.830 (4)	0.709 (3)	−0.333 (2)	0.095 (12)	
H33A	0.8950	0.7709	−0.3691	0.114*	
C34	0.647 (4)	0.699 (3)	−0.362 (2)	0.104 (14)	
C35	0.552 (3)	0.615 (3)	−0.303 (2)	0.086 (10)	
H35A	0.4266	0.6094	−0.3194	0.103*	
C36	0.646 (3)	0.539 (3)	−0.218 (2)	0.073 (9)	
H36A	0.5822	0.4805	−0.1790	0.088*	
C37	0.532 (6)	0.776 (3)	−0.456 (3)	0.19 (2)	
H37A	0.4100	0.7730	−0.4467	0.285*	
H37B	0.5330	0.7275	−0.5278	0.285*	
H37C	0.5814	0.8746	−0.4507	0.285*	

### (II) (S)-(+)-Dichlorido[1-(4-methylphenyl)-N-(pyridin-2-ylmethylidene)ethylamine-κ2N,N']mercury(II) Atomic displacement parameters (Å^2^)

**Table:**

	*U*^11^	*U*^22^	*U*^33^	*U*^12^	*U*^13^	*U*^23^
Hg1	0.0358 (6)	0.0412 (6)	0.0731 (12)	0.0041 (5)	0.0102 (7)	0.0063 (7)
Cl1	0.044 (3)	0.046 (3)	0.104 (7)	0.014 (3)	0.024 (4)	0.022 (4)
Cl2	0.064 (3)	0.036 (3)	0.089 (5)	0.0049 (19)	0.029 (3)	0.008 (3)
N1	0.050 (11)	0.027 (9)	0.046 (14)	0.004 (8)	0.011 (10)	0.005 (9)
C2	0.040 (10)	0.044 (12)	0.08 (2)	0.013 (8)	0.026 (11)	0.008 (12)
C3	0.090 (18)	0.034 (12)	0.06 (2)	0.000 (13)	0.007 (16)	0.001 (12)
C4	0.042 (10)	0.049 (13)	0.08 (2)	0.004 (9)	0.003 (11)	0.039 (12)
C5	0.042 (10)	0.035 (10)	0.056 (15)	−0.006 (7)	0.013 (9)	0.006 (9)
C6	0.025 (9)	0.042 (11)	0.048 (16)	0.007 (8)	0.012 (10)	0.021 (11)
C7	0.046 (11)	0.040 (10)	0.037 (13)	0.015 (8)	0.020 (9)	0.007 (9)
N8	0.044 (10)	0.030 (8)	0.041 (12)	0.005 (7)	0.012 (9)	0.007 (8)
C9	0.048 (10)	0.043 (11)	0.056 (13)	0.007 (8)	0.011 (9)	0.008 (9)
C10	0.070 (9)	0.065 (9)	0.079 (12)	0.030 (7)	0.019 (8)	0.001 (8)
C11	0.072 (15)	0.037 (9)	0.039 (14)	0.007 (9)	0.020 (11)	0.005 (9)
C12	0.084 (17)	0.074 (16)	0.052 (18)	0.017 (12)	0.026 (14)	0.006 (13)
C13	0.14 (3)	0.078 (18)	0.09 (3)	0.026 (17)	0.07 (2)	0.027 (16)
C14	0.13 (3)	0.062 (18)	0.05 (2)	0.022 (18)	0.02 (2)	0.007 (15)
C15	0.09 (2)	0.13 (3)	0.06 (2)	0.03 (2)	−0.016 (17)	−0.01 (2)
C16	0.047 (13)	0.078 (16)	0.06 (2)	0.006 (11)	0.000 (12)	0.004 (14)
C17	0.19 (3)	0.15 (3)	0.06 (2)	0.09 (2)	0.05 (2)	0.05 (2)
Hg2	0.0386 (6)	0.0432 (7)	0.0746 (12)	0.0031 (5)	0.0112 (7)	0.0083 (7)
Cl3	0.116 (5)	0.051 (3)	0.094 (6)	−0.024 (3)	0.060 (5)	−0.012 (3)
Cl4	0.042 (3)	0.077 (5)	0.098 (7)	0.019 (3)	0.019 (4)	0.042 (4)
N21	0.032 (9)	0.044 (11)	0.054 (15)	0.001 (8)	0.010 (9)	0.011 (10)
C22	0.054 (11)	0.040 (10)	0.046 (15)	−0.001 (8)	0.024 (10)	−0.004 (10)
C23	0.060 (13)	0.050 (13)	0.048 (17)	−0.004 (10)	0.016 (12)	0.003 (12)
C24	0.062 (13)	0.030 (9)	0.047 (14)	−0.002 (9)	−0.001 (11)	−0.022 (9)
C25	0.024 (8)	0.066 (14)	0.070 (19)	0.008 (9)	0.008 (11)	0.032 (13)
C26	0.044 (12)	0.027 (10)	0.051 (16)	0.008 (9)	0.009 (11)	0.010 (10)
C27	0.023 (9)	0.061 (13)	0.071 (19)	0.007 (9)	0.014 (11)	0.022 (13)
N28	0.043 (11)	0.051 (10)	0.044 (14)	0.008 (8)	0.010 (9)	0.010 (9)
C29	0.065 (12)	0.058 (12)	0.074 (16)	0.018 (10)	0.034 (12)	0.021 (11)
C30	0.116 (13)	0.051 (9)	0.070 (12)	0.033 (8)	0.032 (10)	0.019 (8)
C31	0.055 (13)	0.058 (13)	0.045 (16)	0.010 (10)	0.006 (11)	−0.015 (11)
C32	0.11 (2)	0.043 (12)	0.08 (2)	−0.022 (13)	0.038 (18)	−0.007 (13)
C33	0.19 (4)	0.037 (11)	0.046 (19)	−0.004 (17)	0.01 (2)	0.000 (12)
C34	0.21 (4)	0.059 (19)	0.03 (2)	0.06 (2)	0.00 (2)	−0.006 (16)
C35	0.11 (2)	0.070 (17)	0.08 (2)	0.038 (16)	−0.001 (18)	0.007 (17)
C36	0.09 (2)	0.073 (17)	0.06 (2)	0.026 (15)	0.017 (16)	0.020 (14)
C37	0.36 (5)	0.10 (2)	0.07 (3)	0.09 (3)	−0.05 (3)	−0.014 (19)

### (II) (S)-(+)-Dichlorido[1-(4-methylphenyl)-N-(pyridin-2-ylmethylidene)ethylamine-κ2N,N']mercury(II) Geometric parameters (Å, º)

**Table:**

Hg1—N1	2.32 (2)	Hg2—N21	2.35 (2)
Hg1—N8	2.405 (19)	Hg2—Cl3	2.397 (7)
Hg1—Cl2	2.406 (6)	Hg2—Cl4	2.419 (7)
Hg1—Cl1	2.410 (6)	Hg2—N28	2.443 (19)
N1—C2	1.32 (3)	N21—C26	1.32 (3)
N1—C6	1.37 (3)	N21—C22	1.34 (3)
C2—C3	1.37 (4)	C22—C23	1.40 (3)
C2—H2A	0.9300	C22—H22A	0.9300
C3—C4	1.40 (3)	C23—C24	1.32 (3)
C3—H3A	0.9300	C23—H23A	0.9300
C4—C5	1.35 (4)	C24—C25	1.37 (3)
C4—H4A	0.9300	C24—H24A	0.9300
C5—C6	1.41 (3)	C25—C26	1.37 (3)
C5—H5A	0.9300	C25—H25A	0.9300
C6—C7	1.50 (4)	C26—C27	1.44 (4)
C7—N8	1.28 (3)	C27—N28	1.25 (3)
C7—H7A	0.9300	C27—H27A	0.9300
N8—C9	1.46 (3)	N28—C29	1.51 (4)
C9—C10	1.49 (2)	C29—C31	1.47 (3)
C9—C11	1.57 (3)	C29—C30	1.53 (2)
C9—H9A	0.9800	C29—H29A	0.9800
C10—H10A	0.9600	C30—H30A	0.9600
C10—H10B	0.9600	C30—H30B	0.9600
C10—H10C	0.9600	C30—H30C	0.9600
C11—C16	1.366 (17)	C31—C36	1.371 (17)
C11—C12	1.369 (17)	C31—C32	1.387 (18)
C12—C13	1.364 (19)	C32—C33	1.352 (19)
C12—H12A	0.9300	C32—H32A	0.9300
C13—C14	1.355 (18)	C33—C34	1.36 (2)
C13—H13A	0.9300	C33—H33A	0.9300
C14—C15	1.377 (18)	C34—C35	1.378 (19)
C14—C17	1.532 (18)	C34—C37	1.530 (19)
C15—C16	1.402 (18)	C35—C36	1.400 (18)
C15—H15A	0.9300	C35—H35A	0.9300
C16—H16A	0.9300	C36—H36A	0.9300
C17—H17A	0.9600	C37—H37A	0.9600
C17—H17B	0.9600	C37—H37B	0.9600
C17—H17C	0.9600	C37—H37C	0.9600
			
N1—Hg1—N8	71.3 (7)	N21—Hg2—Cl3	132.8 (5)
N1—Hg1—Cl2	129.7 (5)	N21—Hg2—Cl4	102.2 (5)
N8—Hg1—Cl2	93.7 (5)	Cl3—Hg2—Cl4	121.7 (3)
N1—Hg1—Cl1	99.4 (5)	N21—Hg2—N28	70.1 (7)
N8—Hg1—Cl1	115.1 (5)	Cl3—Hg2—N28	103.1 (5)
Cl2—Hg1—Cl1	129.6 (2)	Cl4—Hg2—N28	114.3 (5)
C2—N1—C6	117 (2)	C26—N21—C22	121 (2)
C2—N1—Hg1	126.5 (17)	C26—N21—Hg2	116.1 (18)
C6—N1—Hg1	116.4 (16)	C22—N21—Hg2	121.9 (16)
N1—C2—C3	126 (2)	N21—C22—C23	118 (2)
N1—C2—H2A	117.2	N21—C22—H22A	121.1
C3—C2—H2A	117.2	C23—C22—H22A	121.1
C2—C3—C4	117 (3)	C24—C23—C22	122 (3)
C2—C3—H3A	121.7	C24—C23—H23A	119.2
C4—C3—H3A	121.7	C22—C23—H23A	119.2
C5—C4—C3	121 (2)	C23—C24—C25	119 (2)
C5—C4—H4A	119.7	C23—C24—H24A	120.7
C3—C4—H4A	119.7	C25—C24—H24A	120.7
C4—C5—C6	118 (2)	C26—C25—C24	120 (2)
C4—C5—H5A	120.9	C26—C25—H25A	120.1
C6—C5—H5A	120.9	C24—C25—H25A	120.1
N1—C6—C5	122 (2)	N21—C26—C25	121 (2)
N1—C6—C7	117 (2)	N21—C26—C27	118 (2)
C5—C6—C7	121 (2)	C25—C26—C27	121 (2)
N8—C7—C6	119 (2)	N28—C27—C26	124 (2)
N8—C7—H7A	120.3	N28—C27—H27A	118.1
C6—C7—H7A	120.3	C26—C27—H27A	118.1
C7—N8—C9	120 (2)	C27—N28—C29	121 (2)
C7—N8—Hg1	115.3 (17)	C27—N28—Hg2	111.8 (17)
C9—N8—Hg1	124.3 (14)	C29—N28—Hg2	127.3 (16)
N8—C9—C10	117.5 (19)	C31—C29—N28	110 (2)
N8—C9—C11	105.0 (18)	C31—C29—C30	115 (2)
C10—C9—C11	115.3 (18)	N28—C29—C30	109.9 (19)
N8—C9—H9A	106.0	C31—C29—H29A	107.2
C10—C9—H9A	106.0	N28—C29—H29A	107.2
C11—C9—H9A	106.0	C30—C29—H29A	107.2
C9—C10—H10A	109.5	C29—C30—H30A	109.5
C9—C10—H10B	109.5	C29—C30—H30B	109.5
H10A—C10—H10B	109.5	H30A—C30—H30B	109.5
C9—C10—H10C	109.5	C29—C30—H30C	109.5
H10A—C10—H10C	109.5	H30A—C30—H30C	109.5
H10B—C10—H10C	109.5	H30B—C30—H30C	109.5
C16—C11—C12	118.3 (16)	C36—C31—C32	117.4 (18)
C16—C11—C9	119.9 (17)	C36—C31—C29	120.0 (19)
C12—C11—C9	121.5 (17)	C32—C31—C29	123 (2)
C13—C12—C11	120.9 (17)	C33—C32—C31	121 (2)
C13—C12—H12A	119.6	C33—C32—H32A	119.4
C11—C12—H12A	119.6	C31—C32—H32A	119.4
C14—C13—C12	121.8 (17)	C32—C33—C34	122 (2)
C14—C13—H13A	119.1	C32—C33—H33A	119.0
C12—C13—H13A	119.1	C34—C33—H33A	119.0
C13—C14—C15	118.3 (17)	C33—C34—C35	118.5 (18)
C13—C14—C17	122.5 (19)	C33—C34—C37	126 (2)
C15—C14—C17	119.2 (19)	C35—C34—C37	116 (2)
C14—C15—C16	119.8 (18)	C34—C35—C36	119.8 (18)
C14—C15—H15A	120.1	C34—C35—H35A	120.1
C16—C15—H15A	120.1	C36—C35—H35A	120.1
C11—C16—C15	120.6 (17)	C31—C36—C35	121.0 (18)
C11—C16—H16A	119.7	C31—C36—H36A	119.5
C15—C16—H16A	119.7	C35—C36—H36A	119.5
C14—C17—H17A	109.5	C34—C37—H37A	109.5
C14—C17—H17B	109.5	C34—C37—H37B	109.5
H17A—C17—H17B	109.5	H37A—C37—H37B	109.5
C14—C17—H17C	109.5	C34—C37—H37C	109.5
H17A—C17—H17C	109.5	H37A—C37—H37C	109.5
H17B—C17—H17C	109.5	H37B—C37—H37C	109.5
			
C6—N1—C2—C3	0 (4)	C26—N21—C22—C23	−2 (4)
Hg1—N1—C2—C3	173 (2)	Hg2—N21—C22—C23	−172.1 (17)
N1—C2—C3—C4	1 (4)	N21—C22—C23—C24	3 (4)
C2—C3—C4—C5	−2 (4)	C22—C23—C24—C25	−3 (4)
C3—C4—C5—C6	3 (4)	C23—C24—C25—C26	3 (4)
C2—N1—C6—C5	0 (3)	C22—N21—C26—C25	2 (4)
Hg1—N1—C6—C5	−173.5 (18)	Hg2—N21—C26—C25	172.8 (18)
C2—N1—C6—C7	177 (2)	C22—N21—C26—C27	−174 (2)
Hg1—N1—C6—C7	4 (3)	Hg2—N21—C26—C27	−3 (3)
C4—C5—C6—N1	−2 (4)	C24—C25—C26—N21	−3 (4)
C4—C5—C6—C7	−179 (2)	C24—C25—C26—C27	173 (2)
N1—C6—C7—N8	−7 (3)	N21—C26—C27—N28	8 (4)
C5—C6—C7—N8	171 (2)	C25—C26—C27—N28	−169 (2)
C6—C7—N8—C9	−180 (2)	C26—C27—N28—C29	173 (2)
C6—C7—N8—Hg1	6 (3)	C26—C27—N28—Hg2	−7 (3)
C7—N8—C9—C10	−20 (3)	C27—N28—C29—C31	−115 (3)
Hg1—N8—C9—C10	154.2 (13)	Hg2—N28—C29—C31	64 (2)
C7—N8—C9—C11	110 (2)	C27—N28—C29—C30	117 (2)
Hg1—N8—C9—C11	−76.1 (19)	Hg2—N28—C29—C30	−63 (3)
N8—C9—C11—C16	75 (3)	N28—C29—C31—C36	−65 (3)
C10—C9—C11—C16	−154 (2)	C30—C29—C31—C36	60 (3)
N8—C9—C11—C12	−99 (3)	N28—C29—C31—C32	113 (3)
C10—C9—C11—C12	32 (3)	C30—C29—C31—C32	−122 (3)
C16—C11—C12—C13	4 (4)	C36—C31—C32—C33	5 (4)
C9—C11—C12—C13	178 (3)	C29—C31—C32—C33	−173 (3)
C11—C12—C13—C14	−6 (4)	C31—C32—C33—C34	−6 (5)
C12—C13—C14—C15	6 (5)	C32—C33—C34—C35	5 (5)
C12—C13—C14—C17	−176 (3)	C32—C33—C34—C37	−176 (3)
C13—C14—C15—C16	−4 (5)	C33—C34—C35—C36	−2 (5)
C17—C14—C15—C16	177 (3)	C37—C34—C35—C36	179 (3)
C12—C11—C16—C15	−3 (4)	C32—C31—C36—C35	−3 (4)
C9—C11—C16—C15	−177 (3)	C29—C31—C36—C35	175 (3)
C14—C15—C16—C11	3 (5)	C34—C35—C36—C31	2 (5)

### (III) (1S,2S,3S,5R)-(+)-Dichlorido[N-(pyridin-2-ylmethylidene)isopinocampheylamine-κ2N,N']mercury(II) Crystal data

**Table:**

[HgCl_2_(C_16_H_22_N_2_)]	*D*_x_ = 1.952 Mg m^−^^3^
*M**_r_* = 513.84	Melting point: 487 K
Monoclinic, *P*2_1_	Mo *K*α radiation, λ = 0.71073 Å
*a* = 10.216 (3) Å	Cell parameters from 72 reflections
*b* = 7.392 (2) Å	θ = 4.9–12.5°
*c* = 23.352 (6) Å	µ = 9.10 mm^−^^1^
β = 97.459 (14)°	*T* = 298 K
*V* = 1748.6 (8) Å^3^	Irregular, colourless
*Z* = 4	0.4 × 0.2 × 0.1 mm
*F*(000) = 984	

### (III) (1S,2S,3S,5R)-(+)-Dichlorido[N-(pyridin-2-ylmethylidene)isopinocampheylamine-κ2N,N']mercury(II) Data collection

**Table:**

Bruker P4 diffractometer	4910 reflections with *I* > 2σ(*I*)
Radiation source: fine-focus sealed tube	*R*_int_ = 0.045
Graphite monochromator	θ_max_ = 26.2°, θ_min_ = 1.8°
ω scans	*h* = −12→9
Absorption correction: part of the refinement model (Δ*F*) (Walker & Stuart, 1983)	*k* = −9→9
*T*_min_ = 0.075, *T*_max_ = 0.405	*l* = −29→29
9195 measured reflections	3 standard reflections every 97 reflections
6573 independent reflections	intensity decay: 5%

### (III) (1S,2S,3S,5R)-(+)-Dichlorido[N-(pyridin-2-ylmethylidene)isopinocampheylamine-κ2N,N']mercury(II) Refinement

**Table:**

Refinement on *F*^2^	Hydrogen site location: inferred from neighbouring sites
Least-squares matrix: full	H-atom parameters constrained
*R*[*F*^2^ > 2σ(*F*^2^)] = 0.057	*w* = 1/[σ^2^(*F*_o_^2^) + (0.0554*P*)^2^ + 18.0971*P*] where *P* = (*F*_o_^2^ + 2*F*_c_^2^)/3
*wR*(*F*^2^) = 0.166	(Δ/σ)_max_ = 0.001
*S* = 1.11	Δρ_max_ = 1.84 e Å^−^^3^
6573 reflections	Δρ_min_ = −1.76 e Å^−^^3^
386 parameters	Extinction correction: SHELXL2014 (Sheldrick, 2015), Fc^*^=kFc[1+0.001xFc^2^λ^3^/sin(2θ)]^-1/4^
1 restraint	Extinction coefficient: 0.0014 (4)
Primary atom site location: structure-invariant direct methods	Absolute structure: Flack *x* determined using 1701 quotients [(*I*^+^)-(*I*^-^)]/[(*I*^+^)+(*I*^-^)] (Parsons *et al.*, 2013)
Secondary atom site location: difference Fourier map	Absolute structure parameter: −0.05 (2)

### (III) (1S,2S,3S,5R)-(+)-Dichlorido[N-(pyridin-2-ylmethylidene)isopinocampheylamine-κ2N,N']mercury(II) Special details

**Table:**

Experimental. ^1^H NMR and ^13^C NMR spectra were recorded on a Varian spectrometer, using CDCl_3_ as solvent and TMS as internal reference. IR spectra were performed on a Perkin-Elmer 283 B or 1420 spectrometer. The FAB spectra were obtained on a JEOL JMS SX 102A mass spectrometer operated at an accelerating voltage of 10 kV. Melting points were measured using an Electrothermal Mel-Temp 3.0 apparatus and are uncorrected.Spectroscopy for ligand*L*^1^: (*S*)-(+)-1-phenyl-*N*-(2-pyridylmethylidene)ethylamine. Yield (95%), light yellow oil. FT-IR: 1658 cm^-1^ (C=N). ^1^H NMR (500 MHz, CDCl_3_): δ 1.58 (*d*, 3H, CHCH_3_), 4.59 (*q*, 1H, CH), 7.17-8.58 (*m*, 9H, Ar), 8.46 (*s*, 1H, HC=N). ^13^C NMR (500 MHz, CDCl_3_): δ 24.81 (CCH_3_), 69.81 (CHCH_3_), 121.70, 124.93, 126.94, 127.24, 128.73, 136.68, 136.74, 149.58, 160.69 (Ar), 155.00 (HC=N). MS-EI *m*/*z* = 210 (*M*^+^). [α]_D_^25^ = +42.0 (*c*=1, CHCl_3_).Spectroscopy for ligand*L*^2^: (*S*)-(+)-1-(4-methylphenyl)-*N*-(2-pyridylmethylidene)ethylamine. Yield (93%), light yellow oil. FT-IR: 1644 cm^-1^ (C=N). ^1^H NMR (500 MHz, CDCl_3_): δ 1.59 (*d*, 3H, CHCH_3_), 2.31 (*d*, 3H, ArCH_3_), 4.58 (*q*, 1H, CH), 7.14-8.64 (*m*, 8H, Ar), 8.44 (*s*, 1H, HC=N). ^13^C NMR (500 MHz, CDCl_3_): δ 21.13 (CCH_3_), 24.50 (ArCH_3_), 69.25 (CHCH_3_), 121.26, 124.45, 126.42, 128.97, 136.24, 136.38, 141.31, 149.06, 159.99 (Ar), 154.57 (HC=N). MS-EI *m*/*z* = 224 (*M*^+^). [α]_D_^25^ = +31.3 (*c*=1, CHCl_3_).Spectroscopy for ligand*L*^3^: (1*S*,2*S*,3*S*,5*R*)-(+)-(2-pyridylmethylidene)isopinocampheylamine. Yield (90%), light yellow oil. FT-IR: 1644 cm^-1^ (C=N). ^1^H NMR (500 MHz, CDCl_3_): δ 1.01-1.29 (*m*, 9H, 3 CH_3_), 1.26-2.42 (*m*, 7H, H-Aliph), 3.60 (*m*, 1H, N-CH), 7.29 (*m*, 1H, Ar), 7.73 (*m*, 1H, Ar), 8.05 (*m*, 1H, Ar), 8.63 (*m*, 1H, Ar), 8.27 (*s*, 1H, HC=N). ^13^C NMR (500 MHz, CDCl_3_): δ 19.77, 23.56, 27.97, 33.76, 35.66, 38.86, 41.61, 43.80, 47.49 (C-Aliph), 70.06 (N-CH), 121.52, 124.40, 136.48, 149.31, 158.71 (Ar), 154.90 (HC=N). MS-EI *m*/*z* =242 (*M*^+^). [α]_D_^25^ = +30.1 (*c*=1, CHCl_3_).Spectroscopy for complex (I): (*S*)-(+)-[1-phenyl-*N*-(2-pyridylmethylidene)ethylamine-*κ*^2^*N*,*N*']-dichloridomercury(II). Yield (81%), colourless crystals. Mp 139-141 °C (dec). FT-IR: 1647 cm^-1^ (C=N). ^1^H NMR (500 MHz, CDCl_3_): δ 1.91 (*d*, 3H, CHCH_3_), 5.08 (*q*, 1H, CH), 7.26-8.67 (*m*, 9H, Ar), 8.53 (*s*, 1H, HC=N). ^13^C NMR (500 MHz, CDCl_3_): δ 22.91(CCH_3_), 67.70 (CHCH_3_), 127.42, 128.30, 128.46, 128.67, 129.37, 139.73, 140.58, 147.74, 158.49 (Ar), 150.31 (HC=N). MS-EI *m*/*z* = 482 (*M*^+^). [α]_D_^25^ = +9.3 (*c*=1, CHCl_3_).Spectroscopy for complex (II): (*S*)-(+)-[1-(4-methylphenyl)-*N*-(2-pyridylmethylidene)ethylamine-*κ*^2^*N*,*N*']-dichloridomercury(II). Yield (75%), colourless crystals. Mp 145-147 °C (dec). FT-IR: 1641 cm^-1^ (C=N). ^1^H NMR (500 MHz, CDCl_3_): δ 1.89 (*d*, 3H, CHCH_3_), 2.36 (*d*, 3H, ArCH_3_), 5.05 (*q*, 1H, CH), 7.21-8.68 (*m*, 8H Ar), 8.50 (*s*, 1H, HC=N). ^13^C NMR (500 MHz, CDCl_3_): δ 21.14 (CCH_3_), 22.93 (ArCH_3_), 67.35 (CHCH_3_), 127.38, 128.26, 128.39, 130.01, 137.53, 138.53, 139.70, 147.79, 158.33 (Ar), 150.32 (HC=N). MS-EI *m*/*z* = 496 (*M*^+^). [α]_D_^25^ = +8.8 (*c*=1, CHCl_3_).Spectroscopy for complex (III): (1*S*,2*S*,3*S*,5*R*)-(+)-[*N*-(2-pyridylmethylidene)isopinocampheylamine-*κ*^2^*N*,*N*']-dichloridomercury(II). Yield (77%), colourless crystals. Mp 214-216 °C (dec). FT-IR: 1641.5 cm^-1^ (C=N). ^1^H NMR (500 MHz, CDCl_3_): δ 1.06-1.39 (*m*, 9H, 3 CH_3_), 1.36-2.57 (*m*, 7H, H-Aliph), 4.15 (*m*, 1H, N-CH), 7.70-7.74 (*m*, 2H, Ar), 8.06-8.10 (*m*, 1H, Ar), 8.69-8.71 (*m*, 1H, Ar), 8.58 (*s*, 1H, HC=N). ^13^C NMR (500 MHz, CDCl_3_): δ 19.86, 23.50, 27.86, 35.04, 35.65, 38.79, 41.43, 43.46, 47.33 (C-Aliph), 70.60 (N-CH), 128.32, 128.36, 139.50, 147.24, 157.14 (Ar), 150.32 (HC=N). MS-EI *m*/*z* = 514 (*M*^+^). [α]_D_^25^ = +22.7 (*c*=1, CHCl_3_).Biological activity of complexes: The antimicrobial activity of the Hg(II)-complexes (I-III) was evaluated against Gram positive (*Staphylococcus aureus*) and Gram negative (*E. coli* and *Pseudomonas aeruginosa*) bacteria and yeast (*Candida albicans*). The antimicrobial activity were assessed by measuring the Inhibitory zone diameters with the Disk Diffusion Test. We used disk of Amikacin 30 µg, Chloramphenicol 30 µg, Cefepime 30 µg and Fluconazole 25 µg (BD) used for *in vitro* susceptibility testing by the agar disk diffusion test procedure of bacterial and fungal pathogens as antimicrobial control (see Table at the end of this section).According to the results, all complexes were found to possess noteworthy antimicrobial activity. Among the compounds analyzed, (I) and (III) show high antimicrobial activity against all strains assessed, mainly Gram positive bacteria and fungi. In general all complexes tested displayed antifungal activity against the strains of Candida albicans.

### (III) (1S,2S,3S,5R)-(+)-Dichlorido[N-(pyridin-2-ylmethylidene)isopinocampheylamine-κ2N,N']mercury(II) Fractional atomic coordinates and isotropic or equivalent isotropic displacement parameters (Å^2^)

**Table:**

	*x*	*y*	*z*	*U*_iso_*/*U*_eq_	
Hg1	0.15484 (10)	0.69493 (14)	0.30398 (5)	0.0738 (3)	
Cl1	−0.0188 (9)	0.5444 (11)	0.3448 (4)	0.101 (3)	
Cl2	0.3813 (7)	0.6602 (13)	0.3012 (4)	0.099 (2)	
N1	0.0120 (17)	0.852 (4)	0.2299 (7)	0.057 (4)	
C2	−0.058 (3)	0.767 (4)	0.1854 (10)	0.070 (7)	
H2B	−0.0456	0.6439	0.1797	0.084*	
C3	−0.149 (2)	0.866 (6)	0.1474 (10)	0.075 (8)	
H3B	−0.1955	0.8097	0.1153	0.090*	
C4	−0.169 (3)	1.038 (4)	0.1573 (11)	0.074 (7)	
H4B	−0.2313	1.1024	0.1328	0.089*	
C5	−0.098 (2)	1.125 (4)	0.2037 (10)	0.068 (6)	
H5B	−0.1087	1.2480	0.2100	0.082*	
C6	−0.010 (2)	1.021 (4)	0.2406 (9)	0.058 (6)	
C7	0.0603 (18)	1.111 (4)	0.2953 (10)	0.069 (7)	
H7A	0.0616	1.2359	0.3007	0.083*	
N8	0.1175 (19)	1.000 (3)	0.3331 (7)	0.057 (4)	
C9	0.169 (3)	1.082 (4)	0.3899 (9)	0.067 (6)	
H9B	0.1537	1.2130	0.3863	0.081*	
C10	0.314 (3)	1.057 (6)	0.4041 (11)	0.094 (10)	
H10A	0.3375	0.9477	0.3839	0.112*	
C11	0.352 (3)	1.024 (5)	0.4676 (11)	0.094 (11)	
H11A	0.4468	1.0201	0.4802	0.113*	
C12	0.276 (5)	0.854 (5)	0.4821 (16)	0.133 (16)	
H12B	0.2531	0.7723	0.4499	0.160*	
H12C	0.3153	0.7901	0.5164	0.160*	
C13	0.167 (4)	0.987 (6)	0.4925 (12)	0.101 (11)	
H13A	0.1164	0.9530	0.5239	0.121*	
C14	0.085 (3)	1.013 (5)	0.4353 (11)	0.090 (9)	
H14B	0.0155	1.0995	0.4393	0.108*	
H14C	0.0445	0.8994	0.4224	0.108*	
C15	0.272 (3)	1.121 (4)	0.5085 (11)	0.073 (7)	
C16	0.389 (5)	1.209 (11)	0.3825 (15)	0.24 (4)	
H16A	0.3714	1.2143	0.3412	0.354*	
H16B	0.4818	1.1908	0.3939	0.354*	
H16C	0.3624	1.3206	0.3986	0.354*	
C17	0.339 (3)	1.110 (5)	0.5709 (12)	0.102 (10)	
H17A	0.3601	0.9860	0.5806	0.153*	
H17B	0.2801	1.1560	0.5963	0.153*	
H17C	0.4184	1.1804	0.5750	0.153*	
C18	0.238 (4)	1.320 (5)	0.4994 (14)	0.102 (10)	
H18A	0.1771	1.3344	0.4648	0.153*	
H18B	0.3169	1.3878	0.4960	0.153*	
H18C	0.1982	1.3646	0.5318	0.153*	
Hg2	0.40926 (11)	0.34137 (13)	0.18895 (4)	0.0716 (3)	
Cl3	0.5827 (10)	0.4884 (12)	0.1462 (4)	0.106 (3)	
Cl4	0.1863 (9)	0.4162 (14)	0.1910 (5)	0.125 (4)	
N21	0.5612 (17)	0.194 (4)	0.2595 (7)	0.058 (4)	
C22	0.640 (3)	0.272 (5)	0.3028 (11)	0.081 (8)	
H22A	0.6283	0.3946	0.3096	0.097*	
C23	0.737 (2)	0.181 (6)	0.3379 (10)	0.077 (7)	
H23A	0.7931	0.2409	0.3663	0.092*	
C24	0.746 (3)	0.002 (5)	0.3290 (11)	0.081 (8)	
H24A	0.8076	−0.0658	0.3529	0.097*	
C25	0.665 (3)	−0.082 (4)	0.2848 (11)	0.072 (7)	
H25A	0.6722	−0.2061	0.2789	0.087*	
C26	0.577 (2)	0.017 (3)	0.2506 (9)	0.055 (5)	
C27	0.5008 (18)	−0.061 (4)	0.1998 (10)	0.062 (6)	
H27A	0.5127	−0.1817	0.1907	0.074*	
N28	0.4178 (16)	0.0339 (17)	0.1675 (8)	0.048 (4)	
C29	0.352 (2)	−0.059 (3)	0.1136 (9)	0.056 (5)	
H29A	0.3740	−0.1885	0.1164	0.067*	
C30	0.409 (2)	0.020 (3)	0.0602 (9)	0.056 (5)	
H30A	0.4472	0.1378	0.0715	0.067*	
C31	0.299 (2)	0.052 (3)	0.0105 (9)	0.052 (5)	
H31A	0.3296	0.0914	−0.0256	0.063*	
C32	0.199 (2)	0.176 (4)	0.0329 (10)	0.065 (6)	
H32A	0.2366	0.2549	0.0642	0.078*	
H32B	0.1457	0.2444	0.0030	0.078*	
C33	0.133 (2)	0.010 (3)	0.0527 (9)	0.058 (5)	
H33A	0.0362	0.0155	0.0494	0.070*	
C34	0.201 (2)	−0.039 (4)	0.1110 (10)	0.063 (6)	
H34A	0.1647	−0.1515	0.1231	0.076*	
H34B	0.1827	0.0542	0.1382	0.076*	
C35	0.188 (2)	−0.086 (3)	0.0030 (9)	0.055 (5)	
C36	0.516 (2)	−0.095 (3)	0.0422 (11)	0.065 (6)	
H36A	0.5636	−0.0280	0.0163	0.097*	
H36B	0.4787	−0.2015	0.0230	0.097*	
H36C	0.5756	−0.1299	0.0756	0.097*	
C37	0.102 (3)	−0.069 (4)	−0.0549 (9)	0.074 (7)	
H37A	0.1526	−0.1007	−0.0853	0.110*	
H37B	0.0718	0.0540	−0.0603	0.110*	
H37C	0.0275	−0.1480	−0.0558	0.110*	
C38	0.217 (2)	−0.286 (4)	0.0113 (11)	0.069 (6)	
H38A	0.2683	−0.3273	−0.0179	0.103*	
H38B	0.1358	−0.3523	0.0084	0.103*	
H38C	0.2662	−0.3056	0.0487	0.103*	

### (III) (1S,2S,3S,5R)-(+)-Dichlorido[N-(pyridin-2-ylmethylidene)isopinocampheylamine-κ2N,N']mercury(II) Atomic displacement parameters (Å^2^)

**Table:**

	*U*^11^	*U*^22^	*U*^33^	*U*^12^	*U*^13^	*U*^23^
Hg1	0.0653 (6)	0.0756 (6)	0.0795 (7)	−0.0004 (5)	0.0054 (4)	0.0036 (6)
Cl1	0.109 (6)	0.070 (4)	0.135 (7)	−0.005 (4)	0.057 (5)	0.009 (4)
Cl2	0.065 (4)	0.119 (7)	0.111 (5)	0.005 (4)	0.011 (4)	−0.022 (5)
N1	0.056 (10)	0.060 (11)	0.054 (9)	0.003 (11)	0.004 (7)	−0.003 (11)
C2	0.072 (15)	0.076 (17)	0.063 (14)	−0.006 (12)	0.007 (12)	−0.019 (11)
C3	0.058 (13)	0.11 (3)	0.055 (12)	0.000 (17)	0.001 (10)	−0.007 (17)
C4	0.072 (16)	0.09 (2)	0.057 (14)	−0.006 (15)	0.002 (12)	0.017 (14)
C5	0.077 (16)	0.072 (16)	0.056 (13)	−0.003 (12)	0.012 (12)	0.011 (12)
C6	0.053 (12)	0.077 (16)	0.045 (11)	−0.002 (11)	0.004 (9)	−0.004 (11)
C7	0.024 (9)	0.11 (2)	0.078 (15)	−0.002 (11)	0.013 (9)	0.008 (14)
N8	0.067 (11)	0.058 (11)	0.045 (9)	0.002 (9)	−0.004 (8)	0.003 (8)
C9	0.084 (17)	0.074 (16)	0.041 (11)	−0.008 (13)	−0.002 (11)	0.000 (10)
C10	0.062 (15)	0.16 (3)	0.058 (14)	−0.008 (18)	0.005 (12)	−0.029 (18)
C11	0.068 (16)	0.15 (3)	0.060 (15)	0.032 (18)	−0.014 (12)	−0.035 (17)
C12	0.22 (5)	0.056 (17)	0.11 (2)	0.01 (3)	−0.04 (3)	0.01 (2)
C13	0.12 (3)	0.14 (3)	0.053 (15)	−0.04 (2)	0.015 (15)	0.003 (17)
C14	0.082 (18)	0.12 (2)	0.070 (16)	−0.017 (18)	0.004 (14)	−0.022 (17)
C15	0.076 (16)	0.083 (17)	0.057 (14)	−0.003 (13)	0.002 (12)	0.001 (12)
C16	0.16 (4)	0.47 (11)	0.07 (2)	−0.18 (6)	0.04 (2)	0.01 (4)
C17	0.11 (2)	0.12 (3)	0.067 (17)	0.01 (2)	−0.008 (17)	0.000 (17)
C18	0.13 (3)	0.07 (2)	0.09 (2)	−0.01 (2)	−0.010 (18)	−0.031 (18)
Hg2	0.0749 (6)	0.0716 (6)	0.0676 (5)	0.0033 (5)	0.0073 (4)	0.0009 (5)
Cl3	0.134 (7)	0.085 (5)	0.112 (6)	−0.006 (5)	0.060 (5)	0.002 (5)
Cl4	0.083 (5)	0.141 (8)	0.146 (8)	0.036 (5)	−0.005 (5)	−0.068 (6)
N21	0.064 (10)	0.066 (11)	0.043 (8)	−0.007 (12)	0.006 (7)	−0.003 (10)
C22	0.087 (19)	0.09 (2)	0.064 (16)	−0.010 (14)	0.013 (14)	−0.006 (13)
C23	0.068 (14)	0.10 (2)	0.057 (13)	−0.006 (18)	−0.002 (11)	0.007 (17)
C24	0.067 (16)	0.10 (2)	0.066 (15)	−0.007 (15)	−0.013 (12)	0.022 (16)
C25	0.072 (16)	0.086 (18)	0.059 (14)	−0.011 (13)	0.010 (12)	0.018 (13)
C26	0.059 (12)	0.058 (13)	0.047 (11)	0.005 (10)	0.001 (9)	0.001 (10)
C27	0.030 (10)	0.078 (15)	0.074 (14)	−0.003 (10)	−0.003 (9)	−0.013 (12)
N28	0.046 (9)	0.006 (6)	0.088 (12)	0.001 (5)	−0.001 (8)	0.000 (6)
C29	0.052 (12)	0.054 (12)	0.060 (13)	−0.005 (9)	0.005 (10)	−0.004 (10)
C30	0.057 (12)	0.054 (12)	0.057 (12)	−0.002 (10)	0.008 (9)	−0.003 (10)
C31	0.050 (11)	0.056 (12)	0.051 (11)	−0.002 (9)	0.012 (9)	0.001 (10)
C32	0.058 (12)	0.073 (15)	0.062 (13)	0.006 (12)	0.004 (10)	0.004 (12)
C33	0.046 (11)	0.073 (15)	0.057 (12)	0.006 (10)	0.013 (9)	0.009 (11)
C34	0.065 (14)	0.073 (16)	0.056 (13)	−0.010 (12)	0.022 (11)	0.005 (11)
C35	0.051 (11)	0.068 (14)	0.047 (11)	0.008 (10)	0.006 (9)	−0.004 (9)
C36	0.048 (11)	0.069 (14)	0.080 (15)	−0.005 (10)	0.014 (11)	−0.007 (11)
C37	0.066 (15)	0.10 (2)	0.052 (13)	−0.017 (14)	0.003 (11)	0.002 (12)
C38	0.071 (14)	0.064 (15)	0.069 (14)	0.001 (13)	0.006 (11)	−0.009 (13)

### (III) (1S,2S,3S,5R)-(+)-Dichlorido[N-(pyridin-2-ylmethylidene)isopinocampheylamine-κ2N,N']mercury(II) Geometric parameters (Å, º)

**Table:**

Hg1—Cl2	2.337 (7)	Hg2—N28	2.332 (13)
Hg1—Cl1	2.395 (7)	Hg2—Cl4	2.350 (9)
Hg1—N8	2.402 (19)	Hg2—N21	2.37 (2)
Hg1—N1	2.41 (2)	Hg2—Cl3	2.403 (8)
N1—C6	1.30 (4)	N21—C22	1.34 (3)
N1—C2	1.34 (3)	N21—C26	1.34 (4)
C2—C3	1.41 (4)	C22—C23	1.37 (4)
C2—H2B	0.9300	C22—H22A	0.9300
C3—C4	1.31 (5)	C23—C24	1.35 (5)
C3—H3B	0.9300	C23—H23A	0.9300
C4—C5	1.38 (4)	C24—C25	1.38 (4)
C4—H4B	0.9300	C24—H24A	0.9300
C5—C6	1.39 (3)	C25—C26	1.34 (3)
C5—H5B	0.9300	C25—H25A	0.9300
C6—C7	1.54 (3)	C26—C27	1.45 (3)
C7—N8	1.29 (3)	C27—N28	1.27 (3)
C7—H7A	0.9300	C27—H27A	0.9300
N8—C9	1.49 (3)	N28—C29	1.51 (3)
C9—C10	1.49 (4)	C29—C34	1.55 (3)
C9—C14	1.53 (4)	C29—C30	1.55 (3)
C9—H9B	0.9800	C29—H29A	0.9800
C10—C16	1.48 (6)	C30—C36	1.49 (3)
C10—C11	1.50 (4)	C30—C31	1.52 (3)
C10—H10A	0.9800	C30—H30A	0.9800
C11—C15	1.51 (4)	C31—C35	1.52 (3)
C11—C12	1.54 (5)	C31—C32	1.52 (3)
C11—H11A	0.9800	C31—H31A	0.9800
C12—C13	1.53 (6)	C32—C33	1.51 (3)
C12—H12B	0.9700	C32—H32A	0.9700
C12—H12C	0.9700	C32—H32B	0.9700
C13—C15	1.48 (4)	C33—C34	1.49 (3)
C13—C14	1.49 (4)	C33—C35	1.53 (3)
C13—H13A	0.9800	C33—H33A	0.9800
C14—H14B	0.9700	C34—H34A	0.9700
C14—H14C	0.9700	C34—H34B	0.9700
C15—C18	1.53 (4)	C35—C37	1.52 (3)
C15—C17	1.53 (4)	C35—C38	1.52 (4)
C16—H16A	0.9600	C36—H36A	0.9600
C16—H16B	0.9600	C36—H36B	0.9600
C16—H16C	0.9600	C36—H36C	0.9600
C17—H17A	0.9600	C37—H37A	0.9600
C17—H17B	0.9600	C37—H37B	0.9600
C17—H17C	0.9600	C37—H37C	0.9600
C18—H18A	0.9600	C38—H38A	0.9600
C18—H18B	0.9600	C38—H38B	0.9600
C18—H18C	0.9600	C38—H38C	0.9600
			
Cl2—Hg1—Cl1	138.3 (3)	N28—Hg2—Cl4	107.3 (5)
Cl2—Hg1—N8	107.8 (5)	N28—Hg2—N21	70.3 (7)
Cl1—Hg1—N8	99.8 (5)	Cl4—Hg2—N21	129.9 (5)
Cl2—Hg1—N1	122.8 (5)	N28—Hg2—Cl3	107.5 (5)
Cl1—Hg1—N1	95.7 (5)	Cl4—Hg2—Cl3	132.1 (4)
N8—Hg1—N1	69.3 (7)	N21—Hg2—Cl3	92.5 (5)
C6—N1—C2	121 (2)	C22—N21—C26	118 (2)
C6—N1—Hg1	115.4 (14)	C22—N21—Hg2	127 (2)
C2—N1—Hg1	123 (2)	C26—N21—Hg2	115.1 (13)
N1—C2—C3	119 (3)	N21—C22—C23	124 (4)
N1—C2—H2B	120.4	N21—C22—H22A	118.1
C3—C2—H2B	120.4	C23—C22—H22A	118.1
C4—C3—C2	120 (3)	C24—C23—C22	117 (3)
C4—C3—H3B	120.1	C24—C23—H23A	121.7
C2—C3—H3B	120.1	C22—C23—H23A	121.7
C3—C4—C5	121 (3)	C23—C24—C25	121 (3)
C3—C4—H4B	119.6	C23—C24—H24A	119.7
C5—C4—H4B	119.6	C25—C24—H24A	119.7
C4—C5—C6	117 (3)	C26—C25—C24	119 (3)
C4—C5—H5B	121.3	C26—C25—H25A	120.4
C6—C5—H5B	121.3	C24—C25—H25A	120.4
N1—C6—C5	122 (2)	N21—C26—C25	122 (2)
N1—C6—C7	120 (2)	N21—C26—C27	117 (2)
C5—C6—C7	118 (2)	C25—C26—C27	121 (2)
N8—C7—C6	115 (3)	N28—C27—C26	121 (2)
N8—C7—H7A	122.7	N28—C27—H27A	119.5
C6—C7—H7A	122.7	C26—C27—H27A	119.5
C7—N8—C9	115 (2)	C27—N28—C29	115.6 (17)
C7—N8—Hg1	118.6 (17)	C27—N28—Hg2	116.8 (14)
C9—N8—Hg1	125.7 (15)	C29—N28—Hg2	126.8 (12)
C10—C9—N8	112 (2)	N28—C29—C34	108.6 (18)
C10—C9—C14	116 (2)	N28—C29—C30	109.0 (17)
N8—C9—C14	108 (2)	C34—C29—C30	113.8 (18)
C10—C9—H9B	106.8	N28—C29—H29A	108.4
N8—C9—H9B	106.8	C34—C29—H29A	108.4
C14—C9—H9B	106.8	C30—C29—H29A	108.4
C16—C10—C9	112 (4)	C36—C30—C31	111.6 (18)
C16—C10—C11	112 (3)	C36—C30—C29	112.1 (19)
C9—C10—C11	111 (2)	C31—C30—C29	111.0 (18)
C16—C10—H10A	107.1	C36—C30—H30A	107.3
C9—C10—H10A	107.1	C31—C30—H30A	107.3
C11—C10—H10A	107.1	C29—C30—H30A	107.3
C10—C11—C15	117 (3)	C35—C31—C32	85.5 (17)
C10—C11—C12	106 (3)	C35—C31—C30	116.7 (18)
C15—C11—C12	85 (3)	C32—C31—C30	106.9 (17)
C10—C11—H11A	114.6	C35—C31—H31A	114.7
C15—C11—H11A	114.6	C32—C31—H31A	114.7
C12—C11—H11A	114.6	C30—C31—H31A	114.7
C13—C12—C11	85 (2)	C33—C32—C31	87.6 (19)
C13—C12—H12B	114.5	C33—C32—H32A	114.0
C11—C12—H12B	114.5	C31—C32—H32A	114.0
C13—C12—H12C	114.5	C33—C32—H32B	114.0
C11—C12—H12C	114.5	C31—C32—H32B	114.0
H12B—C12—H12C	111.6	H32A—C32—H32B	111.2
C15—C13—C14	116 (3)	C34—C33—C32	107.4 (19)
C15—C13—C12	87 (3)	C34—C33—C35	114.1 (19)
C14—C13—C12	106 (3)	C32—C33—C35	85.3 (17)
C15—C13—H13A	114.8	C34—C33—H33A	115.4
C14—C13—H13A	114.8	C32—C33—H33A	115.4
C12—C13—H13A	114.8	C35—C33—H33A	115.4
C13—C14—C9	111 (2)	C33—C34—C29	114.0 (18)
C13—C14—H14B	109.3	C33—C34—H34A	108.7
C9—C14—H14B	109.3	C29—C34—H34A	108.7
C13—C14—H14C	109.3	C33—C34—H34B	108.7
C9—C14—H14C	109.3	C29—C34—H34B	108.7
H14B—C14—H14C	108.0	H34A—C34—H34B	107.6
C13—C15—C11	88 (3)	C31—C35—C37	112.6 (19)
C13—C15—C18	118 (3)	C31—C35—C38	120 (2)
C11—C15—C18	120 (3)	C37—C35—C38	106 (2)
C13—C15—C17	115 (3)	C31—C35—C33	87.0 (17)
C11—C15—C17	112 (2)	C37—C35—C33	114.2 (19)
C18—C15—C17	104 (2)	C38—C35—C33	116 (2)
C10—C16—H16A	109.5	C30—C36—H36A	109.5
C10—C16—H16B	109.5	C30—C36—H36B	109.5
H16A—C16—H16B	109.5	H36A—C36—H36B	109.5
C10—C16—H16C	109.5	C30—C36—H36C	109.5
H16A—C16—H16C	109.5	H36A—C36—H36C	109.5
H16B—C16—H16C	109.5	H36B—C36—H36C	109.5
C15—C17—H17A	109.5	C35—C37—H37A	109.5
C15—C17—H17B	109.5	C35—C37—H37B	109.5
H17A—C17—H17B	109.5	H37A—C37—H37B	109.5
C15—C17—H17C	109.5	C35—C37—H37C	109.5
H17A—C17—H17C	109.5	H37A—C37—H37C	109.5
H17B—C17—H17C	109.5	H37B—C37—H37C	109.5
C15—C18—H18A	109.5	C35—C38—H38A	109.5
C15—C18—H18B	109.5	C35—C38—H38B	109.5
H18A—C18—H18B	109.5	H38A—C38—H38B	109.5
C15—C18—H18C	109.5	C35—C38—H38C	109.5
H18A—C18—H18C	109.5	H38A—C38—H38C	109.5
H18B—C18—H18C	109.5	H38B—C38—H38C	109.5
			
C6—N1—C2—C3	5 (4)	C26—N21—C22—C23	1 (4)
Hg1—N1—C2—C3	173.3 (18)	Hg2—N21—C22—C23	−171.8 (19)
N1—C2—C3—C4	−3 (4)	N21—C22—C23—C24	−4 (4)
C2—C3—C4—C5	2 (4)	C22—C23—C24—C25	3 (4)
C3—C4—C5—C6	−3 (4)	C23—C24—C25—C26	0 (4)
C2—N1—C6—C5	−6 (3)	C22—N21—C26—C25	2 (3)
Hg1—N1—C6—C5	−175.4 (17)	Hg2—N21—C26—C25	176.0 (18)
C2—N1—C6—C7	174 (2)	C22—N21—C26—C27	−174 (2)
Hg1—N1—C6—C7	4 (3)	Hg2—N21—C26—C27	0 (3)
C4—C5—C6—N1	5 (4)	C24—C25—C26—N21	−3 (4)
C4—C5—C6—C7	−175 (2)	C24—C25—C26—C27	173 (2)
N1—C6—C7—N8	−14 (3)	N21—C26—C27—N28	−4 (3)
C5—C6—C7—N8	166 (2)	C25—C26—C27—N28	−180 (2)
C6—C7—N8—C9	−171.4 (18)	C26—C27—N28—C29	176 (2)
C6—C7—N8—Hg1	16 (2)	C26—C27—N28—Hg2	6 (3)
C7—N8—C9—C10	−120 (3)	C27—N28—C29—C34	128 (2)
Hg1—N8—C9—C10	52 (3)	Hg2—N28—C29—C34	−63 (2)
C7—N8—C9—C14	111 (3)	C27—N28—C29—C30	−107 (2)
Hg1—N8—C9—C14	−77 (3)	Hg2—N28—C29—C30	62 (2)
N8—C9—C10—C16	91 (3)	N28—C29—C30—C36	97 (2)
C14—C9—C10—C16	−145 (3)	C34—C29—C30—C36	−141 (2)
N8—C9—C10—C11	−143 (3)	N28—C29—C30—C31	−137.3 (18)
C14—C9—C10—C11	−19 (4)	C34—C29—C30—C31	−16 (3)
C16—C10—C11—C15	92 (4)	C36—C30—C31—C35	89 (2)
C9—C10—C11—C15	−34 (5)	C29—C30—C31—C35	−37 (3)
C16—C10—C11—C12	−175 (4)	C36—C30—C31—C32	−177 (2)
C9—C10—C11—C12	59 (4)	C29—C30—C31—C32	57 (2)
C10—C11—C12—C13	−89 (3)	C35—C31—C32—C33	28.5 (16)
C15—C11—C12—C13	28 (2)	C30—C31—C32—C33	−88.1 (19)
C11—C12—C13—C15	−29 (2)	C31—C32—C33—C34	86 (2)
C11—C12—C13—C14	87 (3)	C31—C32—C33—C35	−28.2 (15)
C15—C13—C14—C9	37 (4)	C32—C33—C34—C29	−54 (3)
C12—C13—C14—C9	−57 (4)	C35—C33—C34—C29	39 (3)
C10—C9—C14—C13	18 (4)	N28—C29—C34—C33	136 (2)
N8—C9—C14—C13	145 (3)	C30—C29—C34—C33	15 (3)
C14—C13—C15—C11	−77 (3)	C32—C31—C35—C37	87 (2)
C12—C13—C15—C11	29 (2)	C30—C31—C35—C37	−166 (2)
C14—C13—C15—C18	46 (4)	C32—C31—C35—C38	−147 (2)
C12—C13—C15—C18	153 (3)	C30—C31—C35—C38	−40 (3)
C14—C13—C15—C17	170 (3)	C32—C31—C35—C33	−28.1 (16)
C12—C13—C15—C17	−83 (3)	C30—C31—C35—C33	79 (2)
C10—C11—C15—C13	77 (3)	C34—C33—C35—C31	−79 (2)
C12—C11—C15—C13	−29 (2)	C32—C33—C35—C31	28.3 (15)
C10—C11—C15—C18	−44 (4)	C34—C33—C35—C37	168 (2)
C12—C11—C15—C18	−150 (3)	C32—C33—C35—C37	−85 (2)
C10—C11—C15—C17	−167 (3)	C34—C33—C35—C38	44 (3)
C12—C11—C15—C17	87 (3)	C32—C33—C35—C38	151 (2)

### Inhibitory zones in biological tests for (I)–(III)

**Table:**

Complex	*C. albicans*	*P. aeruginosa*	*E. coli*	*S. aureus*
(I)	28 mm	22 mm	20 mm	26 mm
(II)	19 mm	15 mm	9 mm	23 mm
(III)	23 mm	11 mm	11 mm	21 mm
Control (CH_2_Cl_2_)	0	0	0	0
Antibiotic	Fluconazol	Amikacin	Cefepime	Chloramphenicol
	30 mm	21 mm	16 mm	29 mm
