# Supplementary material for: Crystal structures of three mercury(II) complexes [HgCl2 L] where L is a bidentate chiral imine ligand
Source: Acta Crystallogr E Crystallogr Commun. 2015 Nov 7;71(Pt 12):1462–6. doi: 10.1107/S2056989015020368 (PMC4719814; doi:10.1107/S2056989015020368)
Supplement: Supplementary file 5 [file e-71-01462-sup5.pdf]

*Special details*

**Experimental**. $^1$H NMR and $^{13}$C NMR spectra were recorded on a Varian spectrometer, using $CDCl_3$ as solvent and TMS as internal reference. IR spectra were performed on a Perkin-Elmer 283 B or 1420 spectrometer. The FAB spectra were obtained on a JEOL JMS SX 102A mass spectrometer operated at an accelerating voltage of 10 kV. Melting points were measured using an Electrothermal Mel-Temp 3.0 apparatus and are uncorrected.
**Spectroscopy for ligand $L^1$**: (*S*)-(+)-1-phenyl-*N*-(2-pyridylmethylidene)ethylamine. **Yield** (95%), **light yellow oil**. FT-IR: 1658 cm$^{-1}$ (C=N). $^1$H NMR (500 MHz, $CDCl_3$): $\delta$ 1.58 (*d*, 3H, $CHCH_3$), 4.59 (*q*, 1H, CH), 7.17-8.58 (*m*, 9H, Ar), 8.46 (*s*, 1H, HC=N). $^{13}$C NMR (500 MHz, $CDCl_3$): $\delta$ 24.81 ($CCH_3$), 69.81 ($CHCH_3$), 121.70, 124.93, 126.94, 127.24, 128.73, 136.68, 136.74, 149.58, 160.69 (Ar), 155.00 (HC=N). MS-EI *m/z* = 210 ($M^+$). $[\alpha]_D^{23}$ = +42.0 (*c*=1, $CHCl_3$).
**Spectroscopy for ligand $L^2$**: (*S*)-(+)-1-(4-methylphenyl)-*N*-(2-pyridylmethylidene)ethylamine. **Yield** (93%), **light yellow oil**. FT-IR: 1644 cm$^{-1}$ (C=N). $^1$H NMR (500 MHz, $CDCl_3$): $\delta$ 1.59 (*d*, 3H, $CHCH_3$), 2.31 (*d*, 3H, $ArCH_3$), 4.58 (*q*, 1H, CH), 7.14-8.64 (*m*, 8H, Ar), 8.44 (*s*, 1H, HC=N). $^{13}$C NMR (500 MHz, $CDCl_3$): $\delta$ 21.13 ($CCH_3$), 24.50 ($ArCH_3$), 69.25 ($CHCH_3$), 121.26, 124.45, 126.42, 128.97, 136.24, 136.38, 141.31, 149.06, 159.99 (Ar), 154.57 (HC=N). MS-EI *m/z* = 224 ($M^+$). $[\alpha]_D^{23}$ = +31.3 (*c*=1, $CHCl_3$).
**Spectroscopy for ligand $L^3$**: (1*S*,2*S*,3*S*,5*R*)-(+)-(2-pyridylmethylidene)isopinocampheylamine. **Yield** (90%), **light yellow oil**. FT-IR: 1644 cm$^{-1}$ (C=N). $^1$H NMR (500 MHz, $CDCl_3$): $\delta$ 1.01-1.29 (*m*, 9H, 3 $CH_3$), 1.26-2.42 (*m*, 7H, H-Aliph), 3.60 (*m*, 1H, N-CH), 7.29 (*m*, 1H, Ar), 7.73 (*m*, 1H, Ar), 8.05 (*m*, 1H, Ar), 8.63 (*m*, 1H, Ar), 8.27 (*s*, 1H, HC=N). $^{13}$C NMR (500 MHz, $CDCl_3$): $\delta$ 19.77, 23.56, 27.97, 33.76, 35.66, 38.86, 41.61, 43.80, 47.49 (C-Aliph), 70.06 (N-CH), 121.52, 124.40, 136.48, 149.31, 158.71 (Ar), 154.90 (HC=N). MS-EI *m/z* =242 ($M^+$). $[\alpha]_D^{23}$ = +30.1 (*c*=1, $CHCl_3$).
**Spectroscopy for complex (I)**: (*S*)-(+)-[1-phenyl-*N*-(2-pyridylmethylidene)ethylamine-$\kappa^2N,N'$]-dichloridomercury(II). **Yield** (81%), colourless crystals. Mp 139-141 °C (dec). FT-IR: 1647 cm$^{-1}$ (C=N). $^1$H NMR (500 MHz, $CDCl_3$): $\delta$ 1.91 (*d*, 3H, $CHCH_3$), 5.08 (*q*, 1H, CH), 7.26-8.67 (*m*, 9H, Ar), 8.53 (*s*, 1H, HC=N). $^{13}$C NMR (500 MHz, $CDCl_3$): $\delta$ 22.91($CCH_3$), 67.70 ($CHCH_3$), 127.42, 128.30, 128.46, 128.67, 129.37, 139.73, 140.58, 147.74, 158.49 (Ar), 150.31 (HC=N). MS-EI *m/z* = 482 ($M^+$). $[\alpha]_D^{23}$ = +9.3 (*c*=1, $CHCl_3$).
**Spectroscopy for complex (II)**: (*S*)-(+)-[1-(4-methylphenyl)-*N*-(2-pyridylmethylidene)ethylamine-$\kappa^2N,N'$]-dichloridomercury(II). **Yield** (75%), colourless crystals. Mp 145-147 °C (dec). FT-IR: 1641 cm$^{-1}$ (C=N). $^1$H NMR (500 MHz, $CDCl_3$): $\delta$ 1.89 (*d*, 3H, $CHCH_3$), 2.36 (*d*, 3H, $ArCH_3$), 5.05 (*q*, 1H, CH), 7.21-8.68 (*m*, 8H Ar), 8.50 (*s*, 1H, HC=N). $^{13}$C NMR (500 MHz, $CDCl_3$): $\delta$ 21.14 ($CCH_3$), 22.93 ($ArCH_3$), 67.35 ($CHCH_3$), 127.38, 128.26, 128.39, 130.01, 137.53, 138.53, 139.70, 147.79, 158.33 (Ar), 150.32 (HC=N). MS-EI *m/z* = 496 ($M^+$). $[\alpha]_D^{23}$ = +8.8 (*c*=1, $CHCl_3$).
**Spectroscopy for complex (III)**: (1*S*,2*S*,3*S*,5*R*)-(+)-[*N*-(2-pyridylmethylidene)isopinocampheylamine-$\kappa^2N,N'$]-dichloridomercury(II). **Yield** (77%), colourless crystals. Mp 214-216 °C (dec). FT-IR: 1641.5 cm$^{-1}$ (C=N). $^1$H NMR (500 MHz, $CDCl_3$): $\delta$ 1.06-1.39 (*m*, 9H, 3 $CH_3$), 1.36-2.57 (*m*, 7H, H-Aliph), 4.15 (*m*, 1H, N-CH), 7.70-7.74 (*m*, 2H, Ar), 8.06-8.10 (*m*, 1H, Ar), 8.69-8.71 (*m*, 1H, Ar), 8.58 (*s*, 1H, HC=N). $^{13}$C NMR (500 MHz, $CDCl_3$): $\delta$ 19.86, 23.50, 27.86, 35.04, 35.65, 38.79, 41.43, 43.46, 47.33 (C-Aliph), 70.60 (N-CH), 128.32, 128.36, 139.50, 147.24, 157.14 (Ar), 150.32 (HC=N). MS-EI *m/z* = 514 ($M^+$). $[\alpha]_D^{23}$ = +22.7 (*c*=1, $CHCl_3$).
**Biological activity of complexes:** The antimicrobial activity of the Hg(II)-complexes (I-III) was evaluated against Gram positive (*Staphylococcus aureus*) and Gram negative (*E. coli* and *Pseudomonas aeruginosa*) bacteria and yeast (*Candida albicans*). The antimicrobial activity were assessed by measuring the Inhibitory zone diameters with the Disk Diffusion Test. We used disk of Amikacin 30 $\mu$g, Chloramphenicol 30 $\mu$g, Cefepime 30 $\mu$g and Fluconazole 25 $\mu$g (BD) used for *in vitro* susceptibility testing by the agar disk diffusion test procedure of bacterial and fungal pathogens as antimicrobial control (see Table at the end of this section).
According to the results, all complexes were found to possess noteworthy antimicrobial activity. Among the compounds analyzed, (I) and (III) show high antimicrobial activity against all strains assessed, mainly Gram positive bacteria and fungi. In general all complexes tested displayed antifungal activity against the strains of Candida albicans.

*Inhibitory zones in biological tests for (I)–(III)*

| Complex | *C. albicans* | *P. aeruginosa* | *E. coli* | *S. aureus* |
|---|---|---|---|---|
| (I) | 28 mm | 22 mm | 20 mm | 26 mm |
| (II) | 19 mm | 15 mm | 9 mm | 23 mm |
| (III) | 23 mm | 11 mm | 11 mm | 21 mm |
| Control ($CH_2Cl_2$) | 0 | 0 | 0 | 0 |
| Antibiotic | Fluconazol | Amikacin | Cefepime | Chloramphenicol |
| | 30 mm | 21 mm | 16 mm | 29 mm |
